# Flexible receptor architectures for anion recognition *via* ionic hydrogen bonding

**DOI:** 10.1039/d6sc04901j

**Published:** 2026-09-29

**Authors:** Anjusha Prakash, Austin R. Sartori, Aco Radujević, Mikhail Zamkov, Pavel Anzenbacher

**Affiliations:** a Department of Chemistry, Bowling Green State University Bowling Green Ohio 43403 USA; b Center for Photochemical Sciences, Bowling Green State University Bowling Green Ohio 43403 USA; c Department of Physics, Bowling Green State University Bowling Green Ohio 43403 USA

## Abstract

Phosphate anions play key roles in biology, chemistry, the environment, and are important industrial raw materials. However, their recognition, binding, and sensing in water are complicated by their structural diversity, multiple protonation states, and strong hydration. Well-pre-organized macrocyclic receptors are used to increase selectivity and affinity by reducing the entropy associated with the adaptation of the receptor to the phosphate guests. However, the synthesis and purification of macrocyclic sensors is difficult, marred by low yields. Linear (non-macrocyclic) receptors and sensors are easy to synthesize and purify, but may display lower overall affinity and selectivity. We explored the utility of simple linear sensors that contain ionic hydrogen-bonding donors, namely ammonium, isothiouronium, and guanidinium, for phosphorus oxyanions. Anion binding was investigated by NMR spectroscopy and X-ray crystallography. Quantitative binding was determined by fluorescence titrations. Titrations revealed the general order of affinity to be H_2_PO_4_^−^ > HAsO_4_^2−^ > H_2_P_2_O_7_^2−^ > ADP^2−^ ≥ ATP^3−^ ≫ AMP^−^, and *K*_assoc_ ∼ 10^4^–10^5^ M^−1^. These sensors could also bind and sense the potentially carcinogenic herbicide glyphosate (*K*_assoc_ ∼ 10^3^ M^−1^). Due to their flexible linear structure, these sensors were cross-reactive and, with their distinct ionic hydrogen bonding, they also displayed partial selectivity that could be leveraged in sensor arrays amenable to high-throughput sensing. High-throughput fluorescence data were analyzed using machine learning, linear discriminant analysis (LDA), and support vector machine (SVM)-based linear regressions to obtain 100% correct classification and quantification. Regression analyses allowed the determination of unknown analyte concentrations. LODs were ∼1.0 µM for phosphate and pyrophosphate, while LODs for ATP and ADP were determined to be 1.6–4.5 µM. Overall, these simple linear sensors, despite not being able to leverage the macrocyclic effect, provide a viable alternative at a fraction of the cost and synthetic effort.

## Abstract 2

Strong phosphate binding in aqueous solution *via* ionic hydrogen bonding.

## Graphical abstract

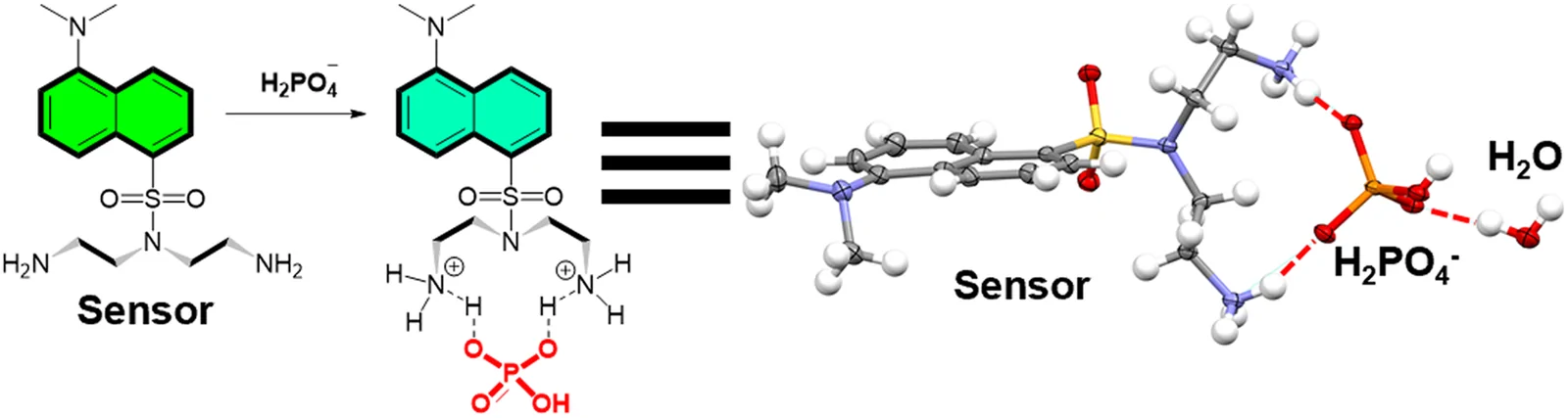

## Introduction

Oxyanions and phosphates play important roles in biology and biochemistry: they maintain electrolyte homeostasis,^1,2^ serve as building blocks for biological phosphorylations,^3,4^ and are limiting nutrients.^5,6^ They are also used as fertilizers^7,8^ and in food additives.^9^ They are used in industry as abrasives,^10^ for pH buffering, water-softening,^11^ and manufacture of LFP batteries.^12^ Phosphate esters are used as additives in flame retardants, plasticizers,^13^ as well as in industrial and aviation fluids.^14,15^ The widespread use, however, carries significant potential for environmental discharge and water-body pollution,^16^ including eutrophication.^17,18^

The importance of phosphates and their applications require effective methods for their sensing, binding, and sequestration. This is, however, particularly challenging due to their extensive hydration^19^ caused by high hydration energies,^20,21^ multiple forms often with varying charges, structural diversity, and frequent presence in mixtures with other anions.^22^ Despite the difficulties, the field of receptors and sensors for anions has made significant progress over recent years.^23–26^

‘Preorganization’ is a powerful tool in supramolecular chemistry that has been used to design artificial host–guest interactions.^27,28^ To limit the negative contribution of the entropic component to molecular recognition associated with the adaptation of hosts to the spatial demands of their guests, macrocyclic hosts are often used because they require lower energy to adopt the conformations required for guest binding.^29^ That said, synthesizing macrocyclic hosts, usually from linear starting materials, is often marred by low yields in cyclization reactions and by the difficulty of obtaining polymeric, macrocyclic, and oligomeric products. In essence, the entropy penalty for host (receptor) adaptation to the guest demand is effectively prepaid by synthesizing the preorganized macrocyclic receptor.^30^ However, the idea of abandoning complex macrocycle syntheses and using simple receptors and sensors is alluring. Recently, we discovered several amine and imine macrocycles 2–4 (Fig. 1) comprising hydrogen-bonding motifs and/or ionic hydrogen bond (IHB) donors. They displayed high affinity for aqueous phosphates and were remarkably effective sensors for oxyanions such as phosphates^31,32^ and phosphonates,^33,34^ including the potentially carcinogenic herbicide glyphosate (RoundUp®).^35,36^

**Fig. 1:**
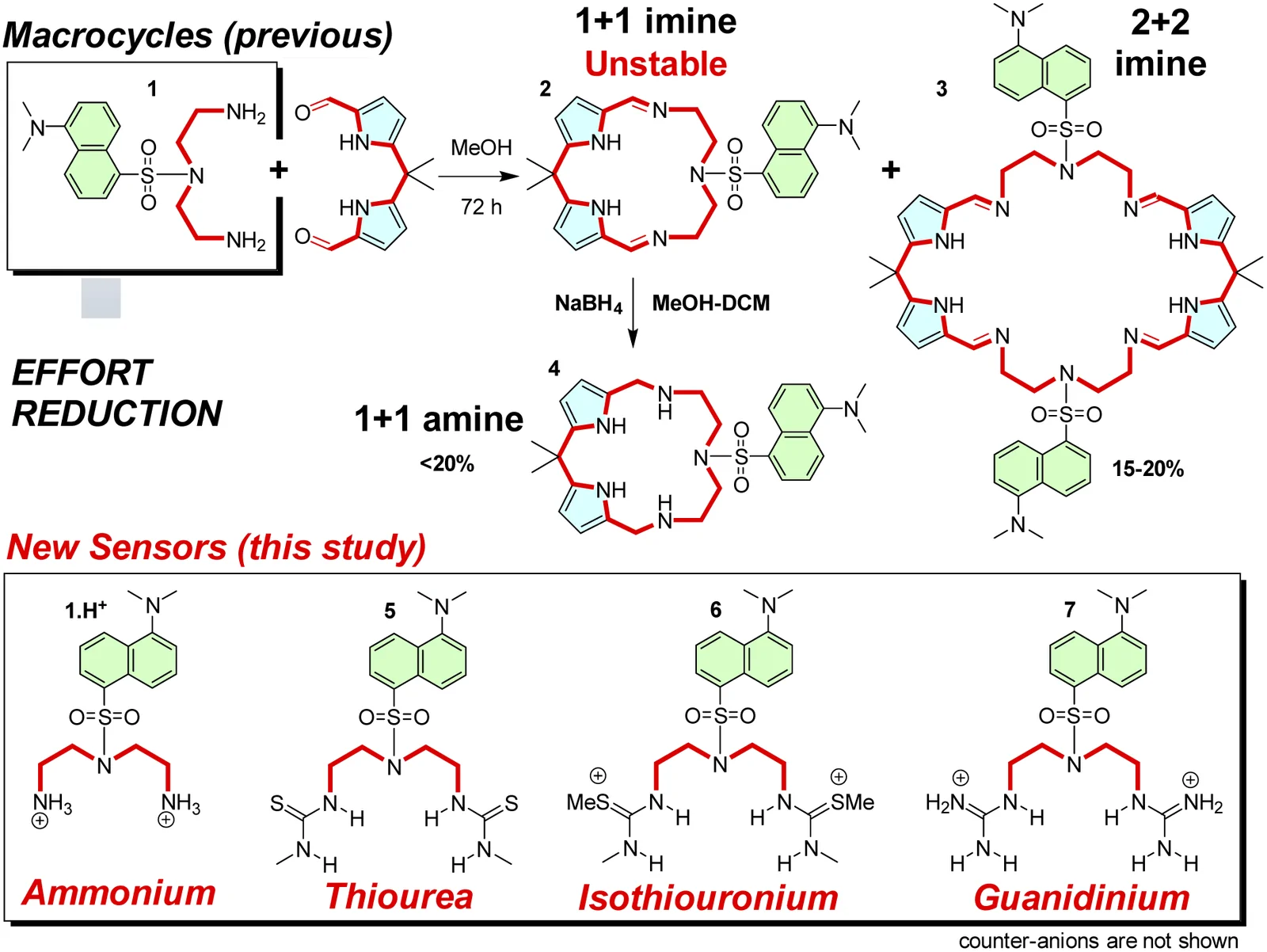
Macrocyclic receptors and sensors 2–4 investigated in our previous studies and the new linear, structurally abbreviated sensors 1, 6, and 7, focused on the utilization of ionic hydrogen bonding.

To investigate the impact of the macrocyclic effect,^37^ we first used the simple diamine fragment 1, a building block for the synthesis of macrocycles 2–4,^31,33^ and its thiourea 5, isothiouronium 6, and guanidinium 7 congeners. Then, we assessed the phosphate-sensing capabilities of these easy-to-make linear sensors, their selectivities and affinities, and compared these to their macrocyclic predecessors.

Notably, receptor moieties in sensors 1, 6, and 7 utilize IHBs for anion binding, whereas thiourea 5 displays the electroneutral hydrogen-bond donors. Compared with the electroneutral hydrogen bonding between molecules (2–10 kcal mol^−1^), which is often ineffective for anion binding in aqueous media, the IHBs are shorter and 10-times stronger (10–40 kcal mol^−1^).^38,39^ Importantly, the IHBs maintain their directionality and, due to their strength, can be harnessed for supramolecular recognition of aqueous analytes. Therefore, compounds 1, 6, and 7 were further investigated for phosphate and anion recognition.

## Results and discussion

### Synthesis

Briefly, diamine 1 was synthesized utilizing our previously reported method.^31^ It was used as a common building block for the synthesis of 5–7, which were prepared as follows: 1 was treated with methyl isothiocyanate to obtain thiourea 5 (in 62% yield),^40^ which was then methylated with iodomethane to obtain isothiouronium iodide 6 (53%).^41^ Finally, 1 was treated with 1H-pyrazole-1-carboxamidine to obtain guanidinium 7 (78%).^42^ The compounds 1 and 5–7 were purified on a silica plug or by crystallization. The exact experimental details are described in the SI.

### Qualitative anion binding studies

With linear sensors 1 and 5–7 in hand, we first investigated their anion binding abilities on a qualitative level using ^1^H-NMR spectroscopy and X-ray crystallography. Fig. 2 shows examples of the isothiouronium sensor 6 with phosphate (Fig. 2, Panel A) and pyrophosphate (Fig. 2, Panel B). As expected, the binding of phosphate anions resulted in concerted shifts of the methyl protons of the isothiouronium moiety H_*j*_ and H_*k*_ located in the close vicinity of the bound anions, confirming binding in a fast-exchange regime. The binding of orthophosphate showed two trends in H_*j*_ and H_*k*_ shifts, indicating two binding events, which were assigned to the formation of a 1 : 1 (H_2_PO_4_^−^ ⊂ 6^2+^) espousing presumably a pincer-like structure and a linear 1 : 2 complex, respectively (Fig. 2, Panel A). Usually, anion binding causes downfield shifts in affected resonances. Here, however, the upfield shift was followed by an opposite trend, and the downfield shift also illuminated the change in the linear receptor conformation as the sensor transitioned from the 1 : 1 complex to the 1 : 2 complex. The complex with pyrophosphate, however, displayed a monotonous trend due to a likely formation of a pincer-like conformation, as observed in the X-ray structure of the orthophosphate HPO_4_^2−^ ⊂ 1^2+^ monohydrate complex (Fig. 3). These stoichiometries were later confirmed in quantitative titration studies. The shifts in the NMR spectra were not due to deprotonation; presumably due to the water (pH = 5.5) needed to maintain the positive charge on the receptors. This hypothesis was also supported by the MS of the complexes (SI S31) and the X-ray structure, which showed the protonated receptor and phosphate along with molecules of water.

**Fig. 2:**
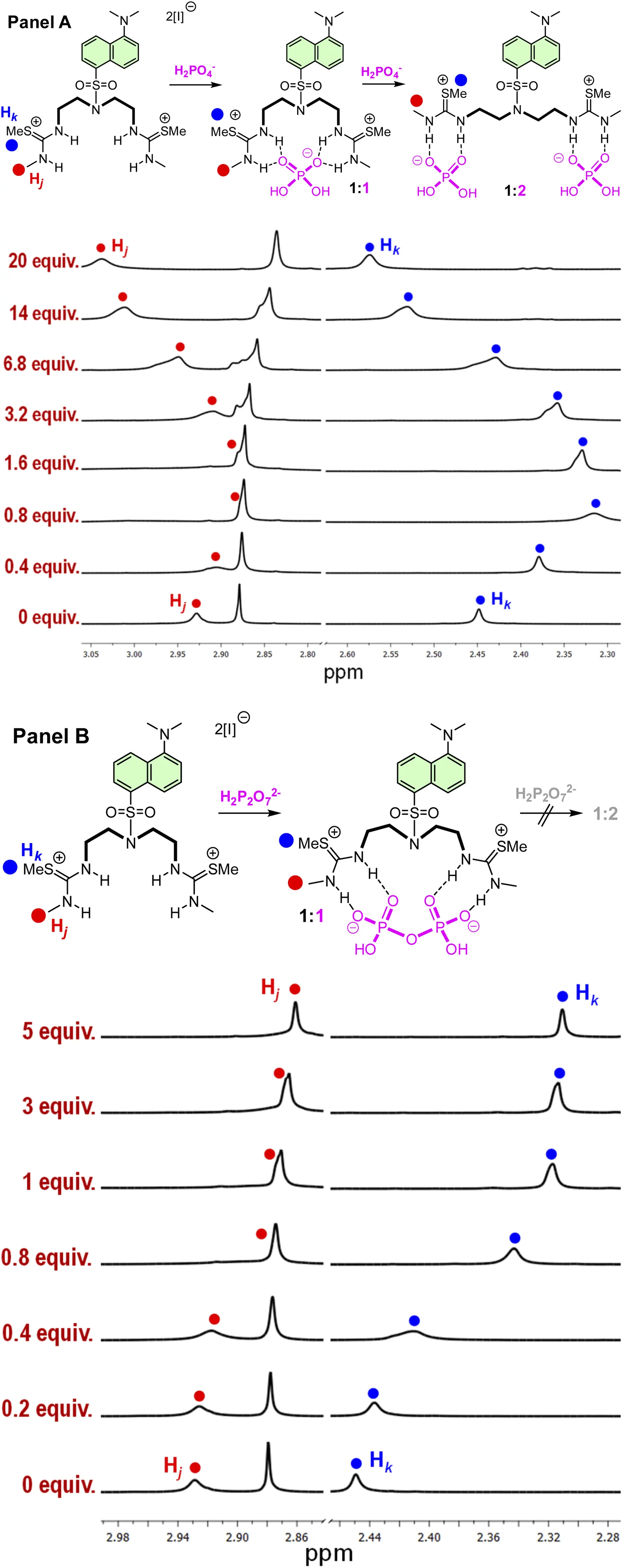
^1^H NMR titration of isothiouronium 6 with phosphates (TBA salts). (Panel A) Titration of 6 with dihydrogen phosphate, H_2_PO_4_^−^. Methyl protons H_*j*_ (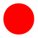) and H_*k*_ (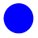) show a downfield chemical shift at low concentrations of Pi and reverses at a higher concentration of Pi, suggesting the possibility of two distinct binding processes. (Panel B) Titration of 6 with hydrogen pyrophosphate, HP_2_O_7_^3−^. Methyl protons H_*j*_ (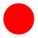) and H_*k*_ (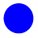) show only a monotonous downfield chemical shift. The details of both titrations are shown in the SI (S10–S11).

**Fig. 3:**
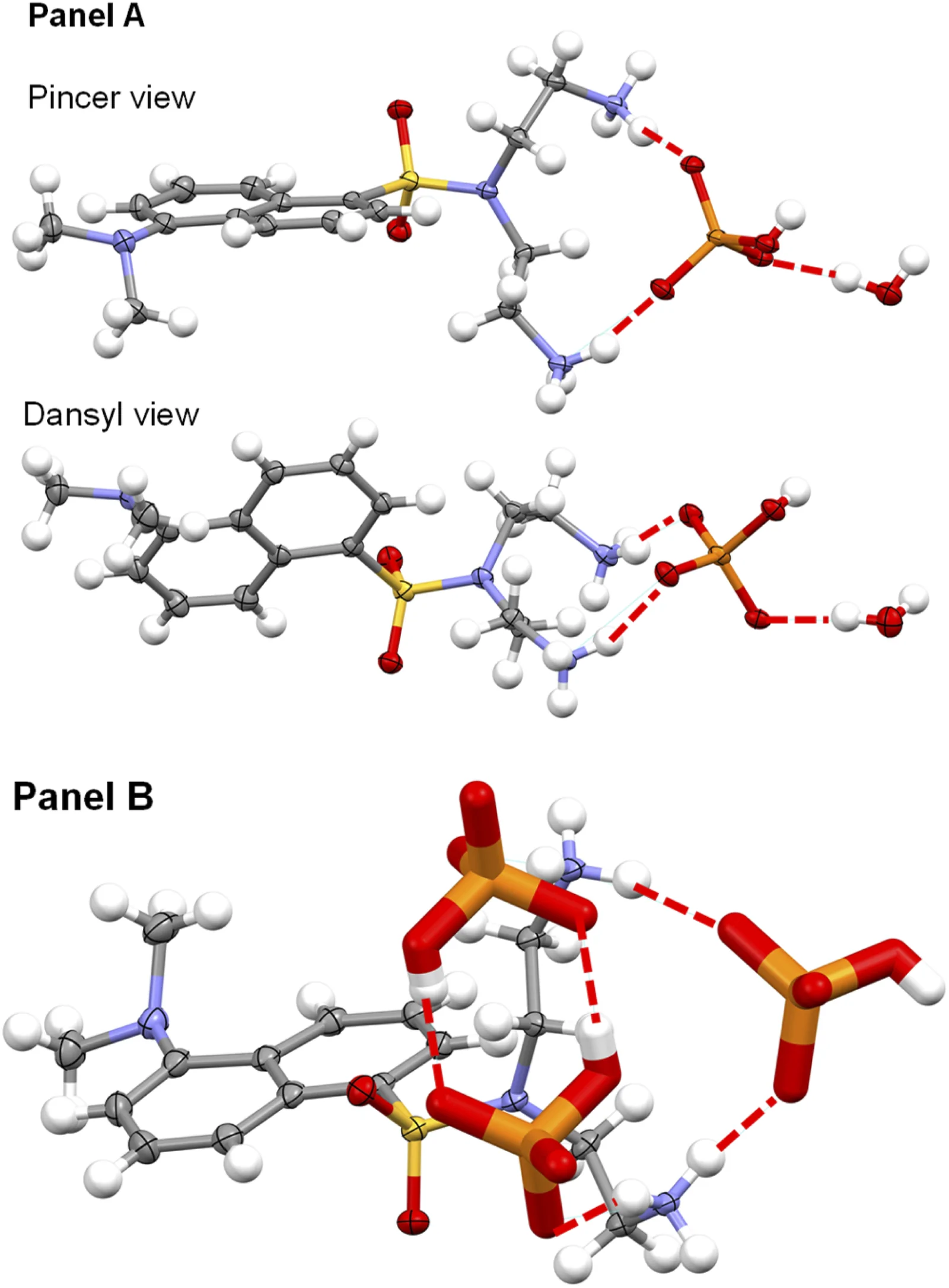
X-ray structure shows the H_2_PO_4_^−^ ⊂ 1^2+^ monohydrate complex and the pincer-like action of the receptor arms (Panel A), as well as the hydrogen-bonded clustering of three orthophosphate anions (highlighted) around the receptor (Panel B).

Further insight into the possible sensor–phosphate complexes could be obtained from the X-ray structure of the complex of 1 with orthophosphate (H_2_PO_4_^−^ ⊂ 1^2+^). The crystals were grown by slow evaporation of acetonitrile from an acetonitrile–water solution of 1. The X-ray structure is shown in Fig. 3. The unit cell showed H_2_PO_4_^−^ ⊂ 1^2+^ monohydrate (Fig. 3, Panel A). The X-ray of the H_2_PO_4_^−^ ⊂ 1^2+^–water complex showed a 1 : 1 (sensor–anion) binding motif, whereas the two arms with positive charges (here, ammonium) displayed a pincer-like action of the receptor moiety with the hydrogen phosphate anion in the center. A water molecule was found to be hydrogen-bonded to the orthophosphate, which is not surprising given the affinity of water for phosphate and because phosphate was added as an aqueous solution (Fig. 3, Panel A). Because the phosphate was added in excess, the sensor was hydrogen-bonded to a cluster of several hydrogen phosphates, suggesting that higher-stoichiometry complexes (1 : 2, 1 : 3, *etc.*) may be encountered, a hypothesis later confirmed by fluorescence titration data (see below). The stoichiometries (*i.e.*, above 1 : 2) would, however, be most likely due to phosphate anion oligomerization due to extensive hydrogen bonding between the anions.^43–47^

The results shown above indicated that the linear receptor based on the diethylene triamine scaffold was a competent anion-binding motif, and that the resulting sensors could bind phosphate anions with 1 : 1 stoichiometry and, quite likely, also 1 : 2 stoichiometry.

### Quantitative anion binding studies

Quantitative studies of the binding affinity of sensors 1, 6, and 7 for anions were performed using fluorescence spectroscopy with dansyl, whose fluorescence intensity and color change upon anion addition. Dansyl is an environmentally sensitive fluorophore that responds to the change in local polarity induced by the bound anion and associated solvate water. The UV-Vis spectrum of dansyl displays an intramolecular charge transfer (ICT) band^48^ at ∼290–420 nm and π–π* absorption below 290 nm. Dansyl fluorescence is due to a combination of two excited states: the blue-green component due to the local excited (LE) state emitting at ∼485 nm, and the ICT excited state emitting at ∼540 nm. The binding of anions in the vicinity and the reorganization of the solvent (water) dipoles have an effect on the two excited states. Different anions, with different solvation, relay these microenvironment changes to dansyl. As a result, the LE and ICT states display changes in intensity, observed as attenuation/amplification and a shift in the LE-ICT composite maximum. Thus, the formation of a static complex anion–sensor complex should induce an anion (analyte)-specific change in fluorescence intensity that can be harnessed for the identification and quantitation of anions (Fig. 4, Panel A). A naked-eye observation of the vials of the solution of the sensors without (Ctrl) and with anions under UV (365 nm) illumination showed weak, yet clearly observable, differences in fluorescence intensity and/or color, which were later quantified by fluorescence titration experiments (Fig. 4, Panel B) to calculate the thermodynamic association constant (*K*_assoc_). The *K*_assoc_ could be calculated from single titration experiments, or from high-throughput measurements performed in 24-replicas using a plate reader, as described in our report^49^ (see SI for details). The calculated thermodynamic *K*_assoc_ constants are listed in Table 1.

**Fig. 4:**
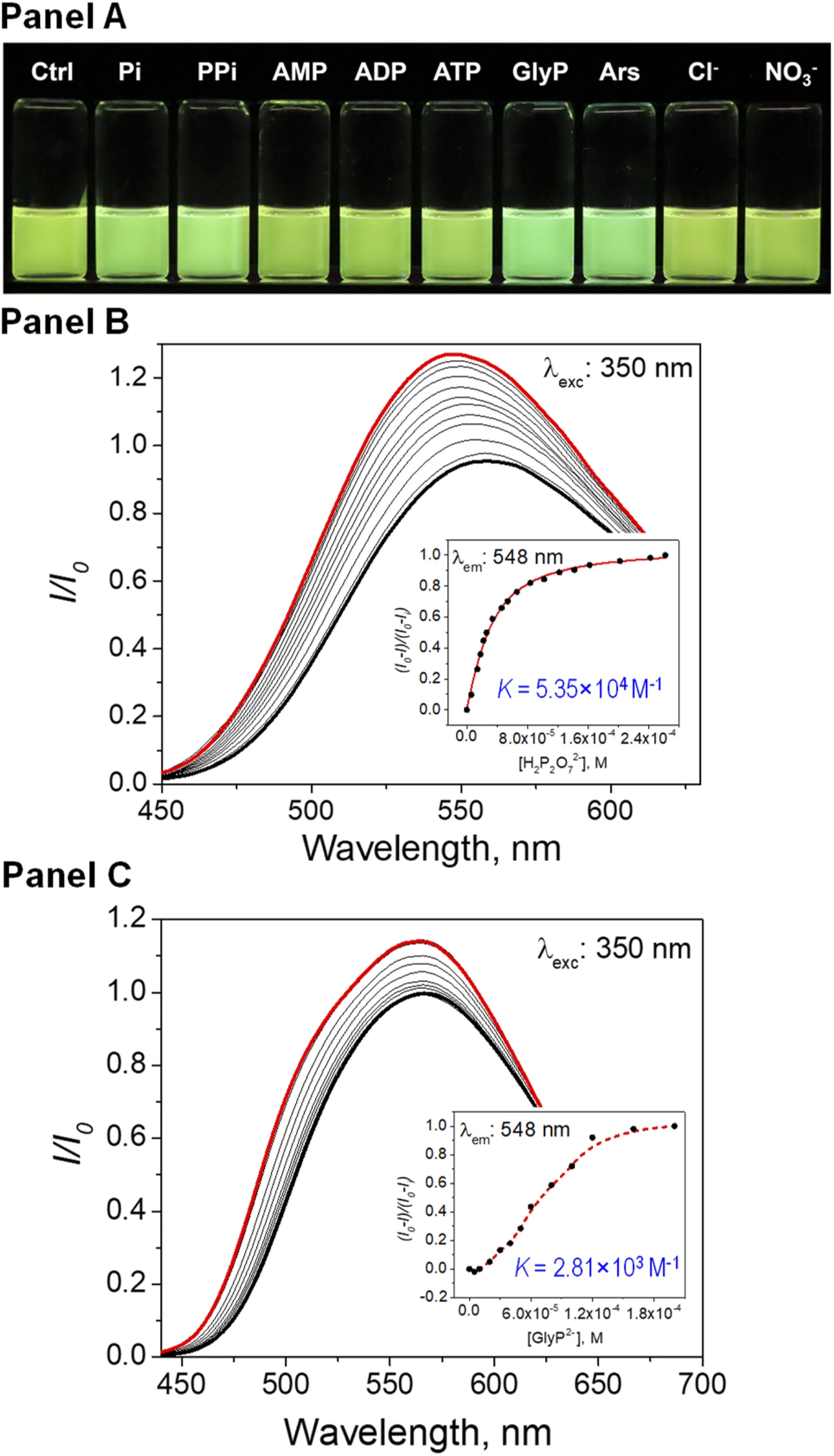
Examples of sensor response to an aqueous anion solution. (Panel A) Sensor 6 (1 µM) in water–DMSO (1 : 9 v/v, pH 5.5) upon incremental addition of phosphates as sodium salts in water (30 equiv.). (Panel B) Fluorescence titration of sensor 6 (15 µM) by disodium pyrophosphate (Panel B) and disodium glyphosate (Panel C), in water. Binding isotherms, measured by fluorescence intensity at *λ*_em_ = 548 nm, are shown as insets. The excitation wavelength was 350 nm in both cases.

**Table 1:** Binding constants (*K*_assoc_, M^−1^) calculated for sensors 1, 6, and 7 and the sodium salts of anionic analytes^a^

Anion/sensor	1 Ammonium	6 Isothiouronium	7 Guanidinium
H_2_PO_4_^−^	3.87 × 10^4^	1.44 × 10^5^	8.00 × 10^3^
		8.82 × 10^4^	
H_2_P_2_O_7_^2−^	5.43 × 10^4^	5.35 × 10^4^^b^	2.81 × 10^4^
HAsO_4_^2−^	2.23 × 10^4^	2.21 × 10^3^	1.14 × 10^4^
		3.14 × 10^4^	
AMP^−^	<100	<100	<100
ADP^2−^	1.50 × 10^4^	1.37 × 10^4^	2.83 × 10^3^
ATP^3−^	2.57 × 10^4^	1.41 × 10^4^	<100
GlyP^2−^	1.54 × 10^3^^b^	2.81 × 10^3^^b^	<100
NO_3_^−^	NR	NR	NR
F^−^	<50	<50	<50
Cl^−^	<50	<50	NR

a *K*_assoc_ values were calculated from the change in fluorescence intensity upon incremental addition of each anion (sodium salts in water) to a 15 µM solution of the sensors in water–DMSO (1 : 9 v/v, pH 5.5). For sensors 1 and 6, the excitation/emission wavelengths were 350/548 nm and, for 7, 400/548 nm. With two exceptions (see SI for values), the fitting errors were <15%. *K*_assoc_ constants were calculated using non-linear least-squares fitting. A previously described high-throughput method was used.^49^ ‘NR’ denotes that an appreciable response was not observed.

b A fluorescence spectrometer was used to acquire full spectra for illustration; see Fig. 4.

Sensors 1, 6, and 7 responded to the addition of aqueous analytes with appreciable but weak changes in fluorescence. The magnitude of changes in fluorescence intensity (Δ*I*) in DMSO–water (9 : 1 v/v) never exceeded 20%. This Δ*I* was weaker than that generally observed in macrocyclic sensors such as 2 and 4 (Fig. 1).^31,33^ However, it was also accompanied by an analyte-specific shift in the LE-ICT composite maximum (ΔFL). This was clearly observable in the fluorescence titrations shown in Fig. 4 (Panels B and C), where we observed fluorescence amplification and the appearance of a blue component as a growing shoulder at ∼500 nm in the glyphosate (GlyP) titration (Fig. 4, Panel C). Δ*I* and ΔFL can be readily measured and provide analyte-specific responses in information-rich data, with significant variance which, in turn, enables accurate classification and quantification of oxyanions, particularly phosphates and related anions (arsenate). Other anions, such as halides, nitrate, carboxylates, or hydrogen sulfate, did not elicit an appreciable response. These results are summarized in Table 1.

Aqueous DMSO was used because the sensors were not fully soluble in pure water, and possible sensor aggregation could hamper analysis. However, they were soluble in 20–30% water in DMSO. The solubility could be adjusted by the choice of counter-anion, which may also compete with analyte binding. Importantly, anionic analytes can be added as solutions in water, which is an important aspect for many applications, because water is the natural medium for most oxyanions. The sensors are meant to be used with a polymer carrier in sensor arrays of polymer-coated optical fiber probes.^21^ Hence, the aqueous solubility of the polymer is not necessary.

As alluded to in the introduction section, the regular hydrogen bonding between electroneutral receptors and sensors is generally weaker (2–10 kcal mol^−1^). The bis-thiourea sensor 5 did not show appreciable binding in water–DMSO, presumably due to competition with water hydrates. However, all sensors (1, 5–7) worked well in pure acetonitrile or DMSO with water content <5%.

Table 1 shows that the sensors were only partially selective (and largely cross-reactive). In fact, each of the sensors 1, 6, and 7 displayed different affinities and cross-reactivities. For example, 6 displayed the highest affinity for most anions but also formed 1 : 1 and 1 : 2 complexes, thereby increasing its dynamic range of responses. Sensor 1, on the other hand, displayed lower overall affinities than 6, but formed only 1 : 1 complexes. Sensor 7 also displayed lower overall affinities but was more selective, providing an appreciable response to only four of the 10 anions tested. This suggests that each sensor could have a unique analytical role if included in a sensor array comprising sensors 1 and 6 as this would generate high-variance data, leading to robust and accurate analysis.

Working with multiple sensors increases data-acquisition complexity. Hence, a high-throughput method using a robotic dispenser and a multi-well fluorescence plate reader, in conjunction with a 1536-well plate platform (working volume = 8 µL), was employed. Herein, the plate reader enabled rapid reading of multiple emission wavelengths to capture the analyte-specific Δ*I* and ΔFL, which could be used further in classification and quantitative analyses. Furthermore, a high number of replicas (24 per sensor per analyte) enabled the construction of a statistically robust model and very accurate analysis, even if small Δ*I* and ΔFL were encountered.

To prove the hypothesis of sensor response complementarity, we subjected the recorded data to linear discriminant analysis (LDA).^50,51^ LDA is a pattern-recognition method that utilizes machine learning to perform multivariate analysis of the data, and classification of the data points into classes corresponding to each of the anions tested. The graphical output then allows for a simpler interpretation of the observed changes in fluorescence into groups (clusters), each corresponding to a different anion (qualitative LDA) or different concentrations of the same analytes (quantitative LDA). The LDA analysis (Fig. 5) clearly showed that the sensor array differentiated between eight aqueous phosphate and glyphosate analytes (Pi, PPi, AMP, ADP, ATP, GlyP, arsenate, chloride), and a control (water). Even the weak responses from analytes such as chloride or AMP were all classified with 100% accuracy. Anions such as nitrate or hydrogen sulfate were not included in the panel because sensors 1 and 6 did not bind to these anions with high affinity and, if included, they would cluster with or near the control. The qualitative LDA (Fig. 5) provided unambiguous confirmation that the spectral behavior was analyte-specific and provided sufficient information (variance) to allow differentiation among the anions.

**Fig. 5:**
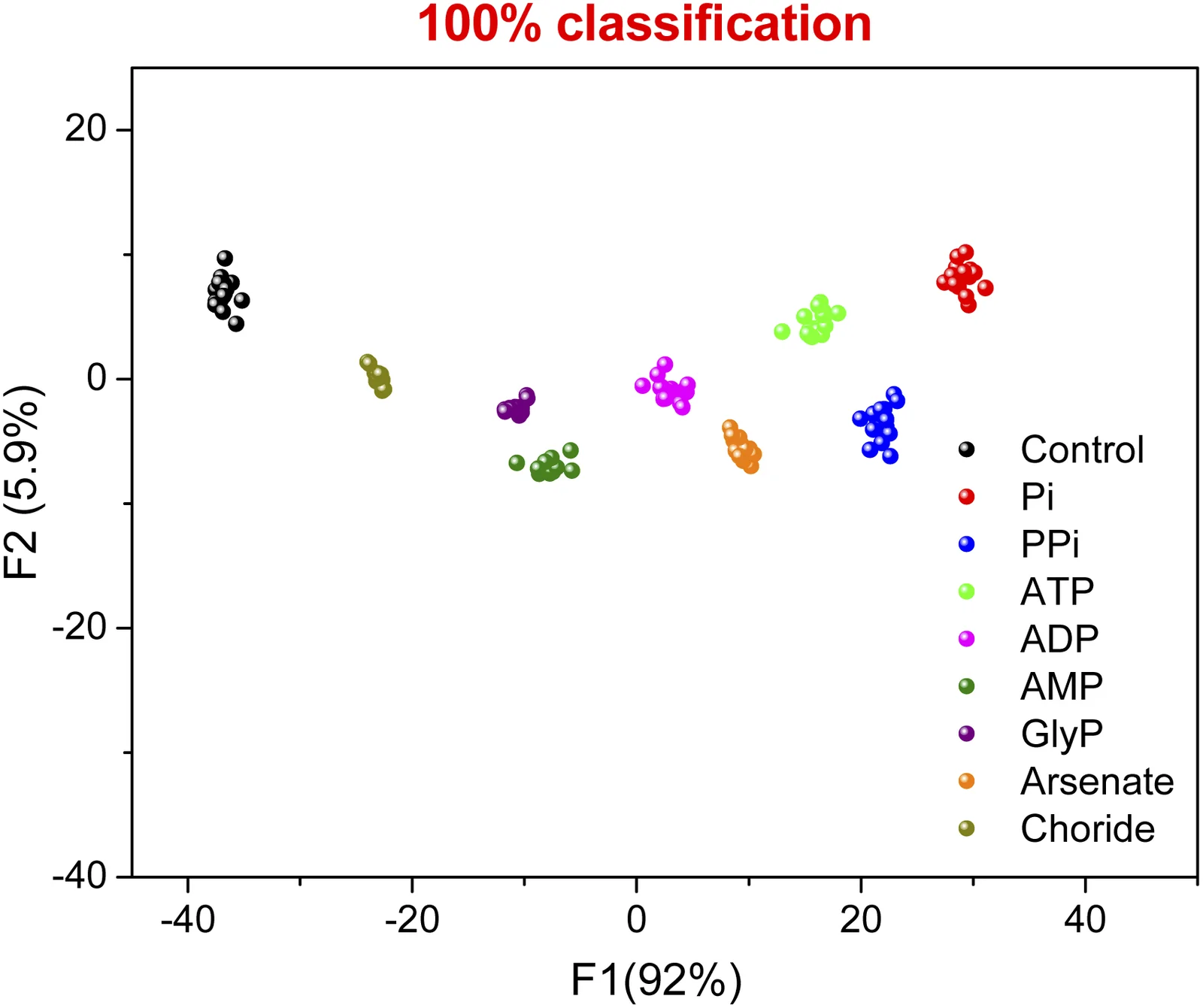
Qualitative LDA shows that the sensor array composed of 1 and 6 (15 µM) achieves perfect discrimination of the eight analytes, each in 20 replicas (total of 180 data points), and assignment to all classes with 100% correct classification. Measured in water–DMSO (1 : 9 v/v, pH 5.5) upon addition of phosphates as sodium salts in water (3 equiv.).

The sensor array composed of 1 and 6 also allowed for quantitative analysis and determination of unknown concentrations of the analytes. This is shown in the examples of quantitative analyses of orthophosphate (Pi) and pyrophosphate (PPi) (Fig. 6, Panel A), as well as ATP and ADP as they occur in biological dephosphorylations of ATP (to ADP and Pi) (Fig. 6, Panel B).

**Fig. 6:**
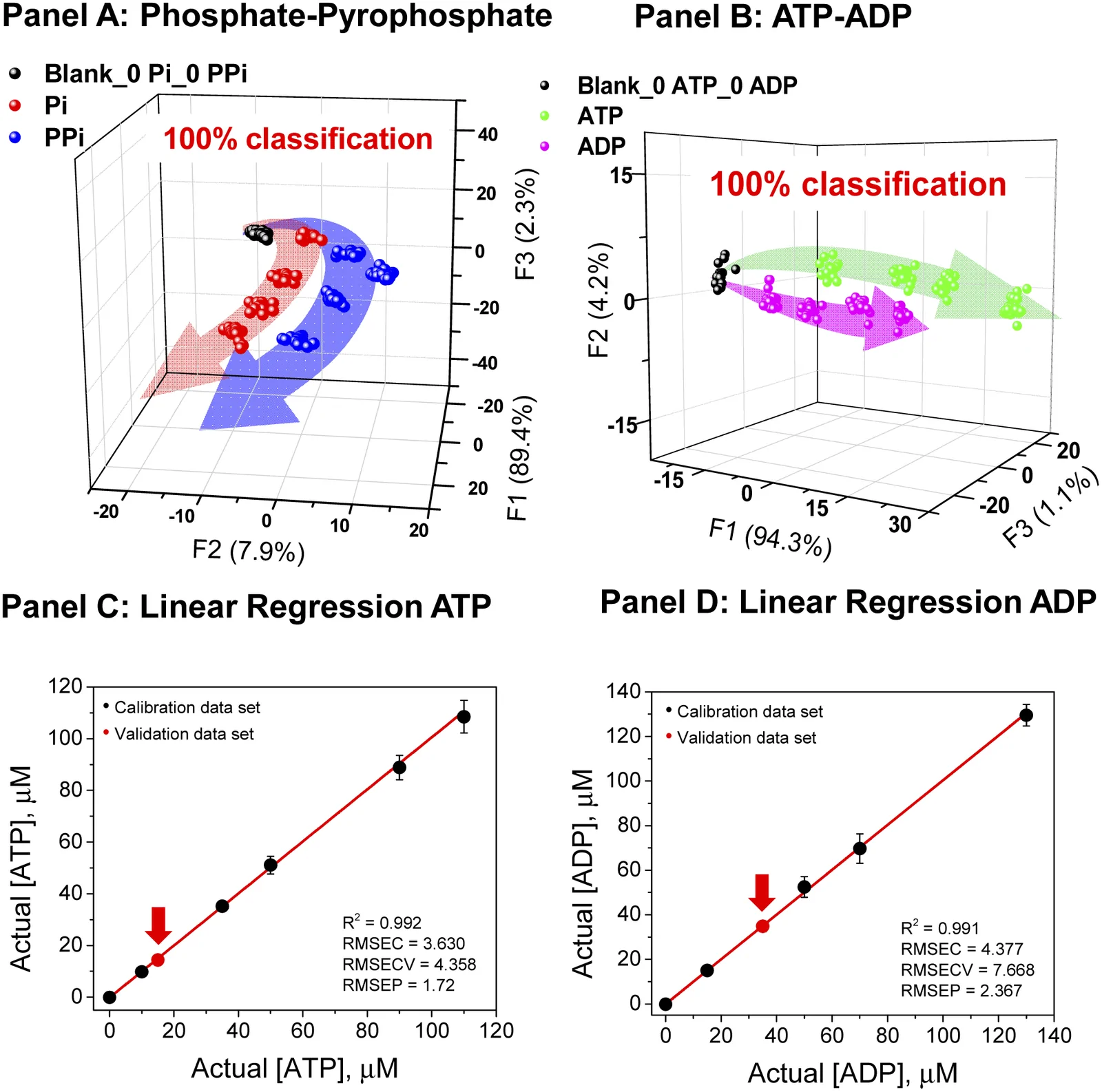
Quantitative analyses using LDA and SVM linear regressions. (Panel A) Quantitative LDA of the orthophosphate (Pi) and pyrophosphate (PPi), concentrations of 0–130 µM. (Panel B) Quantitative LDA of ATP and ADP, concentrations of 0–110 µM and 130 µM, respectively. (Panel C) SVM quantitative linear regression analysis of the concentration of ATP. An unknown ATP concentration shown as a red dot (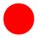) at 15 µM was predicted with 1.7% error. (Panel D) SVM regression analysis of ADP concentrations shows the determination of an unknown ADP concentration (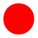) at 35 µM with an error of 2.3%. Measured in water–DMSO (1 : 9 v/v, pH 5.5) upon incremental addition of phosphates as sodium salts in water (see SI).

To illustrate the potential practical utility of the array for quantitative sensing of anions in water, we selected four analytes and paired them based on their similarity and because they are frequently found together: phosphate (Pi) and pyrophosphate (PPi), and ATP and ADP. Despite their similarity, the array gave a distinctive cluster corresponding to the analyte type and concentrations. A clear isotherm-like evolution of each analyte and its corresponding concentration clusters resulted in a smooth trend going from the lowest to the highest concentration. This indicated a predictable pattern in the array responses, confirming that it could differentiate between phosphate concentrations, a feature that may be leveraged in further analyses of the phosphate of interest. Fig. 6 (Panels C and D) shows the results of the quantitative analysis utilizing support vector machine (SVM) linear regression.^52,53^ The SVM regression developed calibration models to predict the behavior of the data corresponding to the unknown concentrations of the analytes (red dot 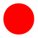 and arrow in Fig. 6, Panels C and D). The unknown concentrations were predicted correctly with errors (root mean square error for prediction, RMSEP) of only 1.7% and 2.3% for ATP and ADP, respectively. This result attested to the excellent ability of the array comprising 1 and 6 to quantify ATP and ADP levels in samples of unknown concentration.

The LOD/LOQ were calculated from the high-throughput data (see SI for details). LODs were determined as follows: for 1, Pi = 0.94 µM, PPi = 1.03 µM, ATP = 3.03 µM, ADP = 4.47 µM; for 6, LODs were Pi = 0.99 µM, PPi = 0.68 µM, ATP = 3.57 µM, ADP = 1.60 µM. These data compared favorably with the data obtained for macrocyclic congeners 2 and 3 (Fig. 1).

## Conclusions

We confirmed the hypothesis that the linear receptors and sensors based on a simple dansyl-labeled diethylene triamine framework were competent sensors for oxyanions, namely phosphates. These ammonium, isothiouronium, and guanidinium compounds were obtained in high yields at a fraction of the cost and effort required for their macrocyclic counterparts. The anion binding by these linear sensors was investigated by NMR, X-ray, and fluorescence spectroscopy.

When compared with the macrocyclic congeners, the linear receptors displayed a lower affinity for anions, presumably due to forgoing the macrocyclic effect. This also incurs an energy penalty associated with the entropy cost of conformational transitions from their linear free forms to the pincer-like chelate. The advantage is that the linear receptors exhibit increased solubility in aqueous DMSO, with up to 30% water compared with the previously prepared macrocyclic congeners. This increased solubility allows a direct comparison with the macrocyclic hosts, which display solubility in the range of 15–20% water in DMSO. This increased water compatibility in linear sensors is presumably due to the higher proportion of polar ionic moieties, ammonium, isothiouronium, and guanidinium. The linear sensors also displayed 1 : 1 and, in the case of isothiouronium sensor 6, 1 : 2 binding modes for small anions such as orthophosphate and arsenate, thereby increasing their dynamic range of responses. The observed binding affinity order was H_2_PO_4_^−^ > HAsO_4_^2−^ > H_2_P_2_O_7_^2−^ > ADP^2−^ ≥ ATP^3−^ ≫ AMP^−^. Interestingly, phosphonomethyl glycine (potentially carcinogenic herbicide glyphosate) was also found to interact with the sensor (*K*_assoc_ = ∼10^3^ M^−1^). In essence, the small size of the diethylene triamine receptor favors smaller anions such as phosphate, arsenate, or pyrophosphate, even though the higher negative charge in phosphates, such as ATP, also imparts reasonable affinity (*K*_assoc_ = ∼10^4^ M^−1^).

The environmentally sensitive dansyl fluorophore changes the fluorescence intensity and spectral shift, providing an analyte-specific response to anions. Three sensors with complementary binding profiles were used to establish a sensor array that utilized a combination of cross-reactivity and selectivity for key analytes to generate information-rich responses, which were analyzed by LDA and SVM regression analyses to enable unambiguous classification of nine analytes and quantification of orthophosphate, pyrophosphate, ADP, and ATP, aimed at biological assays. The particular strength of these simple, easy-to-make sensors lies in high-throughput analyses, where the different ionic hydrogen bonding moieties provide an additional layer of variance that machine learning algorithms can leverage.

In general, the linear receptors and sensors displayed lower affinity compared with the macrocyclic congeners. This was seen in the values of association constants as well from the corresponding LDAs (SI, Table S1 and Fig. S32).

Taken together, these results suggest that sensors based on a small linear diethylene triamine receptor are comparable with larger, more expensive sensors that utilize sophisticated (but expensive) macrocyclic structures at a fraction of the cost. These macrocyclic receptors and sensors are likely to maintain their superiority in certain applications in which a strategically important analyte is being addressed. The excellent binding properties, inexpensive and modular synthesis, and the fact that they are highly amenable to high-throughput applications make these linear sensors promising candidates for future applications.

## Author contributions

AP performed the synthesis, fluorescence and NMR titrations, high-throughput measurements and wrote the SI. ARS contributed to high-throughput measurements, NMR experiments, and calculations of association constants. AR performed the first synthesis of sensor 1 and crystallized the H_2_PO_4_^−^ ⊂ 1^2+^ complex. MZ and PA conceptualized the research, developed the graphics, and wrote the manuscript with input from all authors. All authors have given approval to the final version of the manuscript.

## Conflicts of interest

There are no conflicts of interest to declare.

## Supplementary Material

SC-017-D6SC04901J-s001

## Data Availability

The data supporting this article have been included as part of the supplementary information (SI). Supplementary information: experimental details, NMR and MS spectra, crystallography, fluorescence titration data, and linear discriminant analysis data, with associated figures and tables. See DOI: https://doi.org/10.1039/d6sc04901j.
