# Supplementary material for: Flexible receptor architectures for anion recognition via ionic hydrogen bonding
Source: Chem Sci. 2026 Sep 29. Online ahead of print. doi: 10.1039/d6sc04901j (PMC13621351; doi:10.1039/d6sc04901j)
Supplement: Supplementary file 1 [file SC-OLF-D6SC04901J-s001.pdf]

Supplementary Information (SI) for Chemical Science.
This journal is © The Royal Society of Chemistry

# Supporting Information

## Flexible Receptor Architectures for Anion Recognition via Ionic Hydrogen Bonding

Anjusha Prakash, Austin R. Sartori, Aco Radujevic, Mikhail Zamkov, Pavel Anzenbacher Jr.

### Table of Contents

General Experimental....2
NMR Measurements....2
UV-Vis Absorption Measurements....2
Fluorescence Measurements....2
High-Throughput (HT) Fluorescence Measurements....2
Experimental Methods and Characterization Data....3
$^1$H NMR Titration Experiments....11
UV-Vis and High-Throughput Titration Experiments....12
UV-Vis and Fluoresence Titration Experiments....17
Qualitative Linear Discriminant Analysis....22
Quantitative Linear Discriminant Analysis....24
Limit of Detection and Limit of Quantitation Determination....27
Complex formation: Evidence from ESI-MS....28
Macrocyclic vs Linear sensors comparison....29
X-ray Crystallography....30
References....32

## General Experimental

Commercially available reagents were used as received from the suppliers without further purification unless otherwise noted. Dichloromethane (DCM), pentane, tetrahydrofuran (THF), N,N-dimethylformamide (DMF), and methanol (MeOH) were obtained from EMD Millipore Corp.; 200 proof ethanol (EtOH) from Pharmco-Aaper; and spectrophotometric grade dimethyl sulfoxide (DMSO) from Thermo Fisher Scientific. The nano-pure water used had a resistivity of 18.2 MΩ·cm. Diethylenetriamine was purchased from Alfa Aesar; phthalic anhydride, N,N-diisopropylethylamine (DIPEA), 4-(dimethylamino)pyridine (DMAP), methyl isothiocyanate, 1H-Pyrazole-1-carboxamidine hydrochloride and aluminium oxide ($Al_2O_3$) from Aldrich Chem. Co.; dansyl chloride from Ambeed, iodomethane and hydrazine monohydrate from Acros Organics and glacial acetic acid from LabChem. The analytes used for titrations were purchased from the following suppliers: Sodium dihydrogen phosphate (Pi) and disodium dihydrogen pyrophosphate (PPi) from Ambeed, Adenosine 5′-triphosphate disodium salt (ATP) from TCI, Adenosine 5′-diphosphate disodium salt (ADP) from VWR, Adenosine 5′-monophosphate disodium salt (AMP) from Fluka, Glyphosate (GlyP) from ChemScene and Sodium arsenate dibasic from Alfa Aesar. Na₂SO₄ was used as drying agent in work-up procedures. Analytical thin layer chromatography (TLC) was performed on glass-backed pre-coated silica gel plates with $F_{254}$ fluorescent dye (0.25 mm, Sorbent Technologies) and visualized under UV light (254/365 nm), or by exposure to iodine vapor, KMnO₄, or ninhydrin with subsequent heating.

MALDI (Matrix Assisted Laser Desorption/Ionization) was performed using Shimadzu Biotech AXIMA Performance MALDI TOF/TOF Mass Spectrometer where dithranol was used as a matrix. For neutral/positively charged molecules, reflectron High-Resolution mode were used. High-Res MALDI spectra were obtained by internally spotting the solution of DIT (dissolved in freshly distilled THF), low-mass peptide mix as a calibrant mixture (dissolved in $CH_3CN$: $H_2O$: TFA = 50%: 50%: 0.1%), and desired sample (dissolved in THF). Column chromatography was performed using silica gel (pore size 60 Å, 230-400 mash), 50-200 $\mu$m received from Dynamic Adsorbent Inc.

**NMR Measurements.** NMR spectra were recorded using Bruker AVANCE spectrometer operating at 500 MHz for $^1$H, and 125 MHz for $^{13}$C NMR. Chemical shifts are given in parts per million from $Me_4Si$ in $CDCl_3$, and $D_2O$. The signal multiplicity was described as follows: s (singlet), d (doublet), t (triplet), m (multiplet). Coupling constants (J) are reported in hertz (Hz). Deuterated solvents were purchased from Cambridge Isotope Laboratories.

**UV-Vis Absorption Measurements.** UV-Vis absorption spectra and titration experiments were performed using an Agilent Cary 60 double-beam UV-Vis spectrophotometer over a wavelength range of 200-700 nm. The measurement parameters were set as follows: an average time of 0.1 ms, a data interval of 1 nm, and a scan rate of 600 nm/s. All measurements were conducted using quartz cuvettes with dimensions of 1 × 1 cm.

General protocol: 1.0 mM stock solution sensors were prepared in MeOH and diluted to a working concentration of 30 $\mu$M in 10% $H_2O$ in DMSO solution (pH 5.5 for **1**, **6**, and **7** and pH 9.5 for **5** at r.t). 20 mM stock solution of analytes (sodium salt of anions) were prepared in nano-pure water (pH 5.5 at r.t). After each analyte addition, the absorption spectrum was recorded. All absorbance titration experiments were conducted in duplicate to ensure reproducibility.

**Fluorescence Measurements.** Steady-state fluorescence emission measurements were performed using a single-photon counting spectrofluorometer (Edinburgh Analytical Instruments, FL/FS 920). All solutions were prepared and measured in quartz cuvettes with dimensions of 1 × 1 cm. Excitation wavelengths were selected either at the isosbestic point or at wavelengths where minimal absorbance change was observed based on UV-Vis titration data. The excitation and emission slit widths were set to 1.0 nm. Manufacturer-supplied correction files were applied to all data sets. Fluorescence emission scans were acquired between 400 nm and 700 nm, with a step size of 2.0 nm and a dwell time of 0.8 second per point.

General protocol: 1.0 mM stock solution of sensors were prepared in MeOH and diluted to a working concentration of 15 $\mu$M in 10% $H_2O$ in DMSO solution (pH 5.5 for **1**, **6**, and **7** and pH 9.5 for **5** at r.t). 20 mM stock solution of analytes (sodium salt of anions) were prepared in nano-pure water (pH 5.5 at r.t). After each analyte addition, the emission spectrum was recorded. All fluorescence titration experiments were conducted in duplicate to ensure reproducibility.

**High-Throughput (HT) Fluorescence Measurements.** Automated HT experiments were carried out by a 16-channel microfluidic dispensing robot (Nanodrop Express, BioNex Solutions) with calibration profiles created for each type of solvent ($H_2O$ and DMSO) shortly before use to ensure accuracy of the dispenses. All solutions (sensor and analyte) were dispensed in 1,536 well plate (working volume of 8 $\mu$l) and the plate was centrifuged at 2,000 rpm for 2 minutes. Afterwards, a BMG CLARIOstar microplate reader was used to record the fluorescence intensities from each well at one (or more) wavelengths upon excitating either at the isosbestic point or at wavelengths where minimal absorbance change was observed based on UV-Vis titration data.

General Protocol: A 16.6 $\mu$M stock solution of diamine **1** and isothiouronium **6** were prepared in DMSO (pH 5.5 at r.t) and series of analyte stock solutions ranging from 10 $\mu$M to 4 mM were prepared in nano-pure water (pH 5.5 at r.t). Using the 16-channel microfluidic pipetting robot, 7.2 $\mu$l stock solution of sensor and 0.8 $\mu$l stock solution of analyte (various concentration)

were dispensed into 1,536 well plate (MATRICAL MP111-1-PS, working volume 8.0 $\mu$l). This resulted in final concentrations of 15.0 $\mu$M for sensors and 0-400 $\mu$M for analyte in each well (24 replicas for each data point). After dispensing, the plate was centrifuged, and BMG CLARIOstar microplate reader was used to record the fluorescence intensities from each well. 9 different combinations of excitation and emission channels were used: ex. 320 nm and em. 520 nm, ex. 320 nm and em. 548 nm, ex. 320 nm and em. 558 nm, ex. 350 nm and em. 520 nm, ex. 350 nm and em. 548 nm, ex. 350 nm and em. 558 nm, ex. 400 nm and em. 520 nm, ex. 400 nm and em. 548 nm, ex. 400 nm and em. 558 nm. The resulting fluorescence data (24 replicas) were then subjected to the student's t-test to exclude outliers, resulting in 20 final replicas for each data point. Titration isotherms were obtained by plotting fluorescence intensity (average of the 20 final replicas) vs. concentration of analyte. The binding constant and stoichiometry was evaluated using different fitting models (http://supramolecular.org/, Musketeer 1.10.1 and OriginPro 8.6), and the fits were evaluated using three criteria: first, the overall fit was visually evaluated for how well the fitting model fits experimental data, secondly, binding constants were analyzed with respect to the corresponding fitting errors (lower than 40%) and finally, only the fits that yield random distribution of fit residuals were used. The datasets were then subjected to LDA for qualitative assessment and support vector machine (SVM) algorithms for quantitative evaluation.

## Experimental Methods and Characterization Data

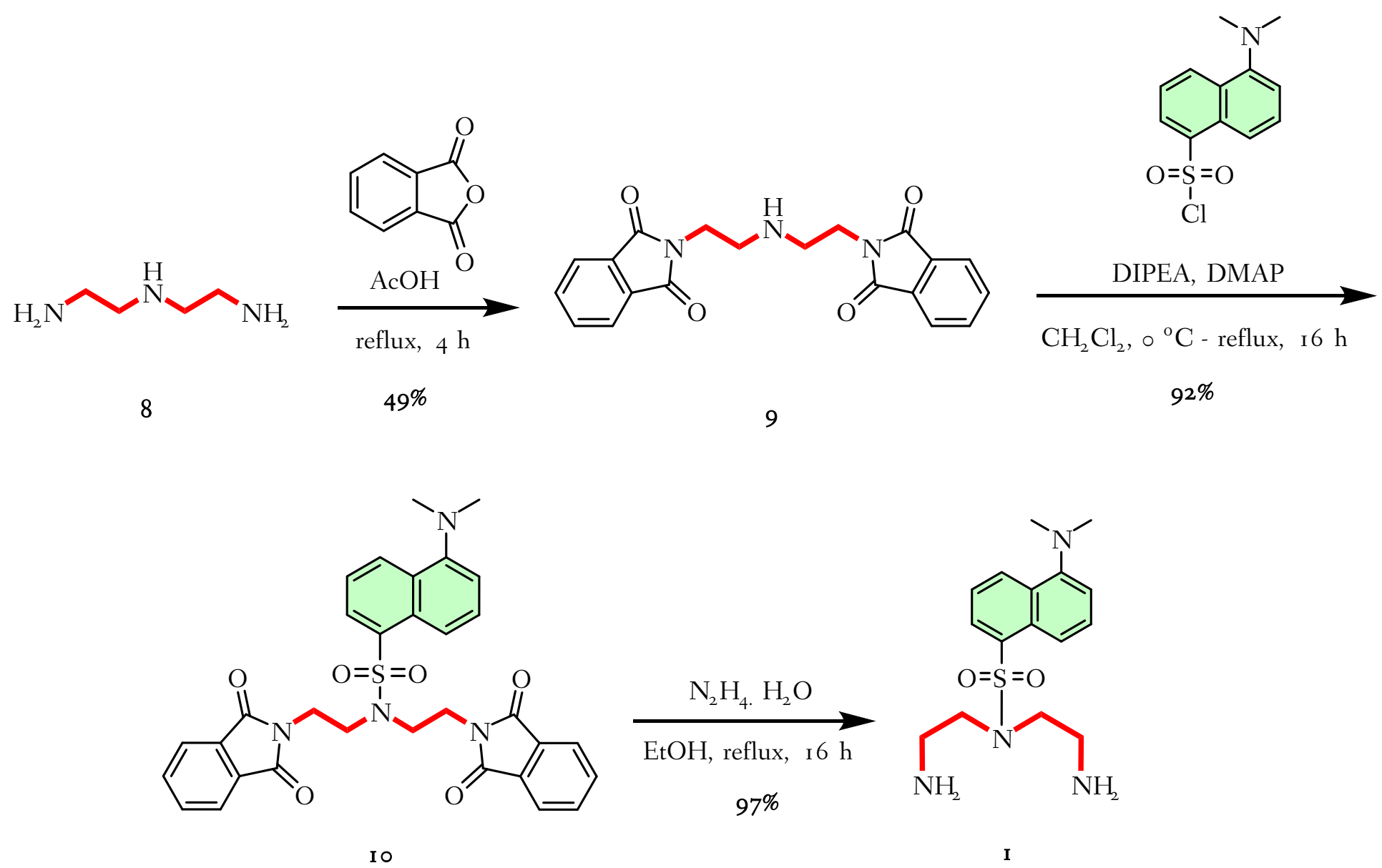


**Scheme S1**. Synthesis of diamine 1.

Compounds **9**, **10** and **1** are previously known and their preparations were adapted from reported procedures without significant modification. Spectroscopic data was consistent with those previously reported.[1]

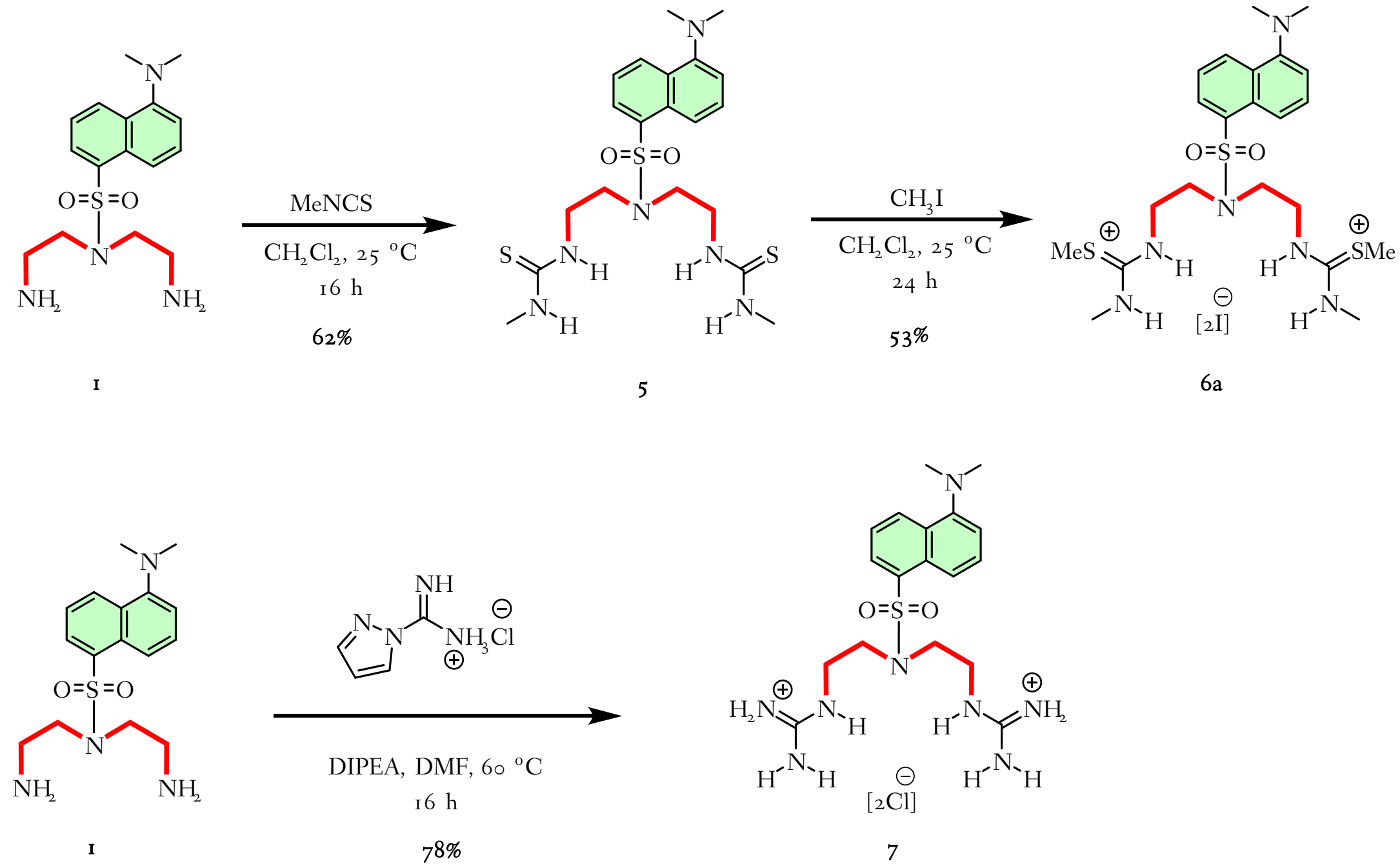


**Scheme S2**. Synthesis of thiourea, isothiouronium and guanidinium derivatives of diamine **1**.

5-(dimethylamino)-N,N-bis(2-(3-methylthioureido)ethyl)naphthalene-1-sulfonamide (Thiourea **5**): To a solution of diamine **1** (0.57 g, 1.70 mmol) in 15 mL of DCM, methyl isothiocyanate (0.25 g, 3.40 mmol) was added and the mixture was stirred at room temperature overnight. Upon completion of the reaction as indicated by TLC, the crude was diluted with 10 mL water and extracted with DCM 3 times. The combined organic layers were dried over sodium sulfate ($Na_2SO_4$), filtered, and concentrated under reduced pressure. The residue was purified by silica plug using 5% MeOH in DCM gradient to afford compound 4 as yellow solid (0.503 g, 62%)[2]; $^1$H NMR (500 MHz, $CDCl_3$) δ 8.56 (d, *J* = 8.5 Hz, 1H), 8.26 (d, *J* = 8.5 Hz, 1H), 7.99 (d, *J* = 7.3 Hz, 1H), 7.55 (dt, *J* = 27.5, 8.0 Hz, 2H), 7.19 (d, *J* = 7.6 Hz, 1H), 6.66 (s, 2H), 6.47 (s, 2H), 3.81 (s, 4H), 3.55 (s, 4H), 2.99 (s, 6H), 2.88 (s, 6H); $^{13}$C NMR (125 MHz, $CDCl_3$) δ 182.67, 152.08, 133.28, 131.02, 130.29, 130.03, 128.84, 128.70, 123.26, 118.78, 115.46, 49.77, 45.41, 44.24, 30.74; HRMS (ESI) m/z: calcd. for $C_{20}H_{30}N_6O_2S_3$ [M+H] 483.1671 found 483.1648 (□□m□= 4.7 ppm).

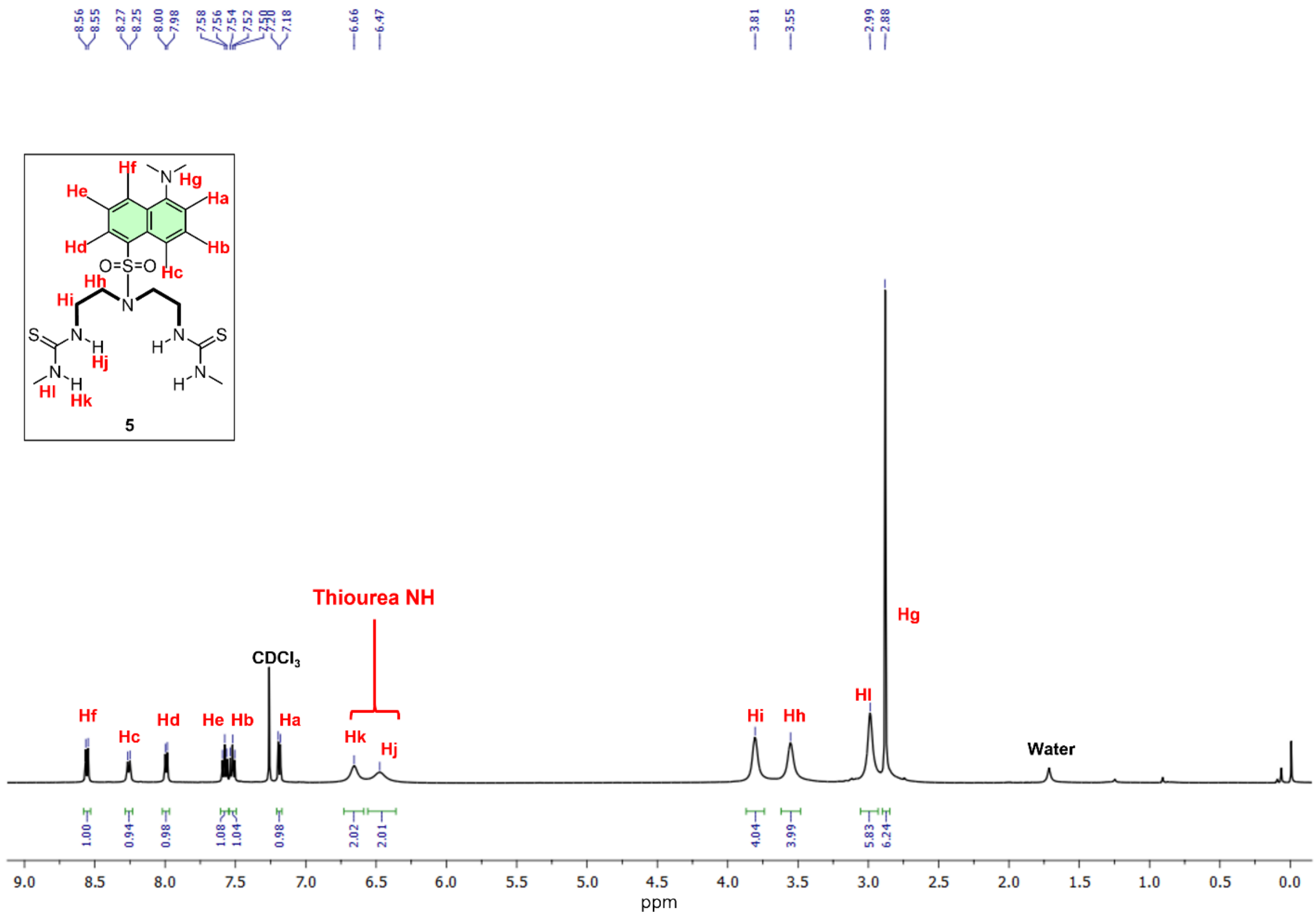


Figure S1: $^{1}$H NMR (500 MHz, $CDCl_3$) of thiourea 5.

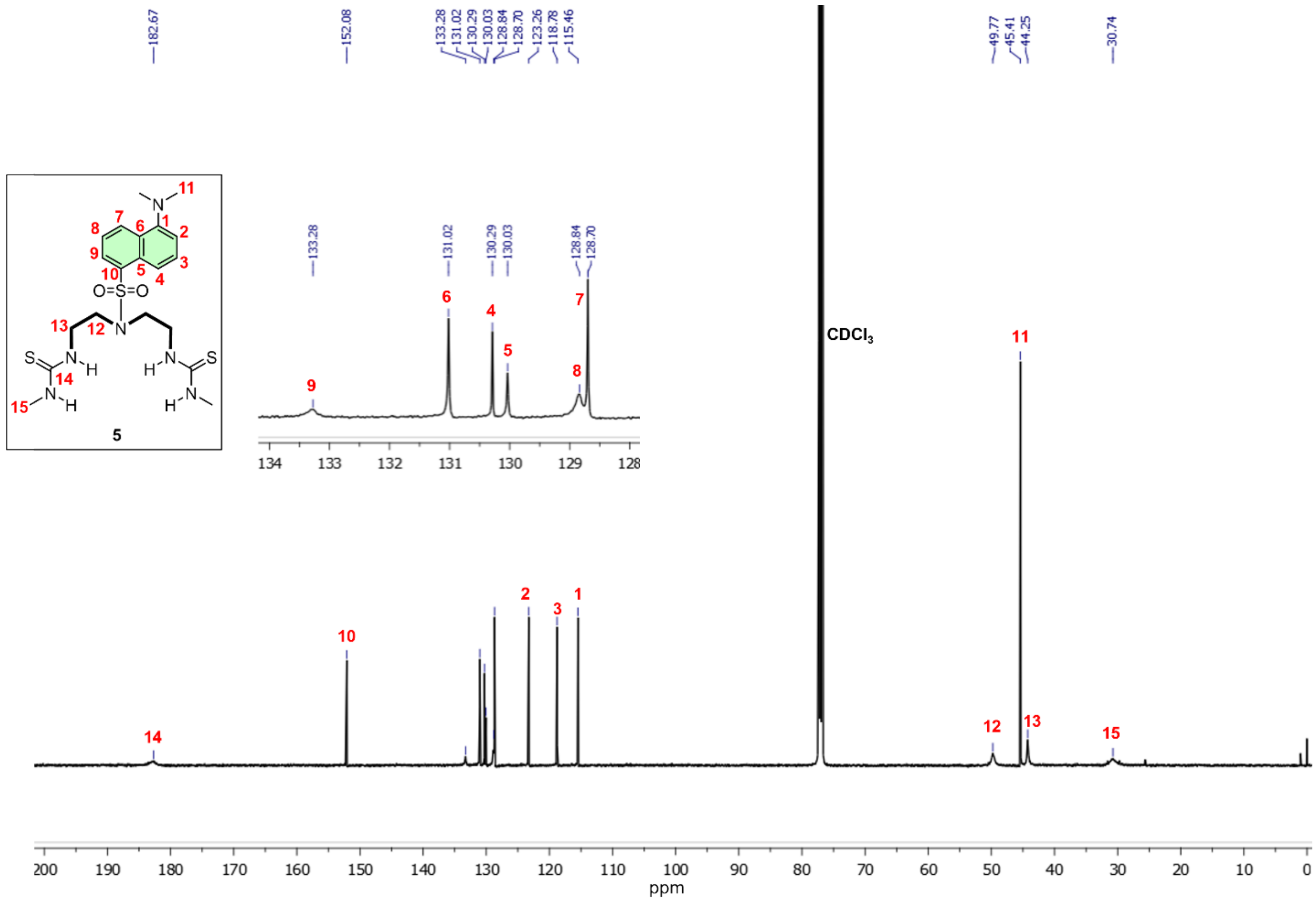


**Figure S2**: $^{13}C$ NMR (125 MHz, $CDCl_3$) of thiourea **5**.

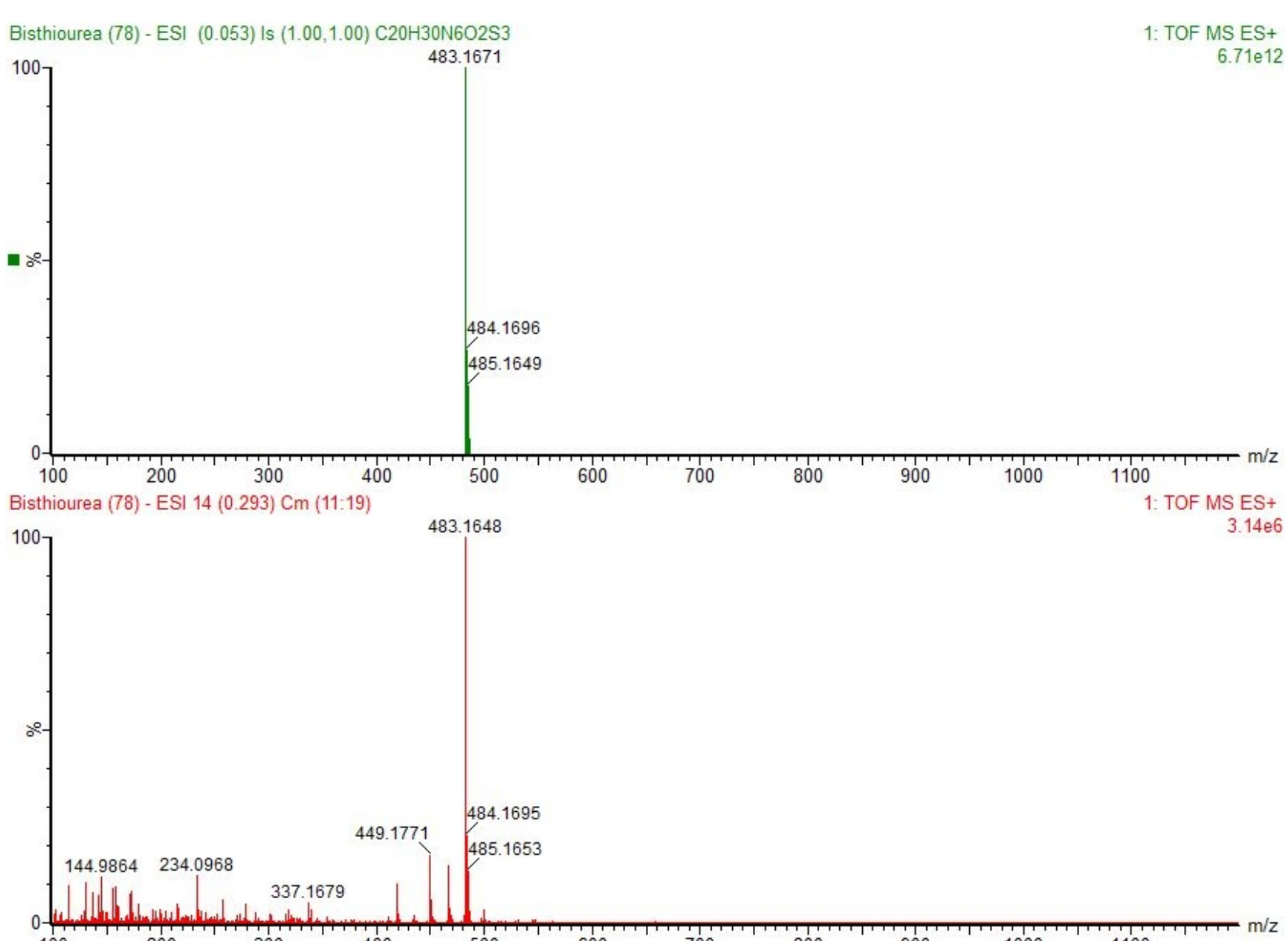


**Figure S3**: HRMS data of thiourea **5**. Top panel shows calculated mass and bottom panel shows observed mass.

1,1'-((((5-(dimethylamino)naphthalen-1-yl)sulfonyl)azanediyl)bis(ethane-2,1-diyl))bis(2,3-dimethylthiouronium iodide) (Isothiouronium **6**): Methyl iodide (1.03 mL, 16.56 mmol) was added dropwise to a solution of thiourea **5** (0.40 g, 0.82 mmol) in DCM (10 mL) at room temperature. The reaction mixture was stirred for 24 hours at room temperature and then concentrated under reduced pressure. The residue was purified by alumina plug using a 6% MeOH in DCM gradient to yield compound 6a as yellow solid (0.333 g, 53%)[3]; $^{1}H$ NMR (500 MHz, $CDCl_3$) δ 8.52 (d, *J* = 8.5 Hz, 1H), 8.30 (d, *J* = 8.7 Hz, 1H), 8.11 (d, *J* = 7.2 Hz, 1H), 7.57 – 7.53 (m, 1H), 7.49 (dd, *J* = 8.3, 7.5 Hz, 1H), 7.17 (d, *J* = 7.5 Hz, 1H), 3.60 – 3.56 (m, 4H), 3.53 (d, *J* = 5.3 Hz, 4H), 2.90 (s, 6H), 2.87 (s, 6H), 2.39 (s, 6H).); $^{13}C$ NMR (125 MHz, $CDCl_3$) δ 156.52, 151.82, 134.79, 130.39, 130.20, 130.09, 128.83, 128.28, 123.18, 119.40, 115.31, 49.11, 45.43, 32.70, 29.72, 14.04; HRMS (ESI) m/z: calcd. for $C_{22}H_{36}N_6O_2S_3$ [M-H] 511.1984 found 511.1980 (□□m□= 0.8 ppm) and [M/2] 256.1030 found 256.1032 (□□m□= 0.8 ppm)

Tetrafluoroborate salt 6 of isothiouronium 6a was then prepared through anion metathesis using saturated aqueous solution of $NaBF_4$.

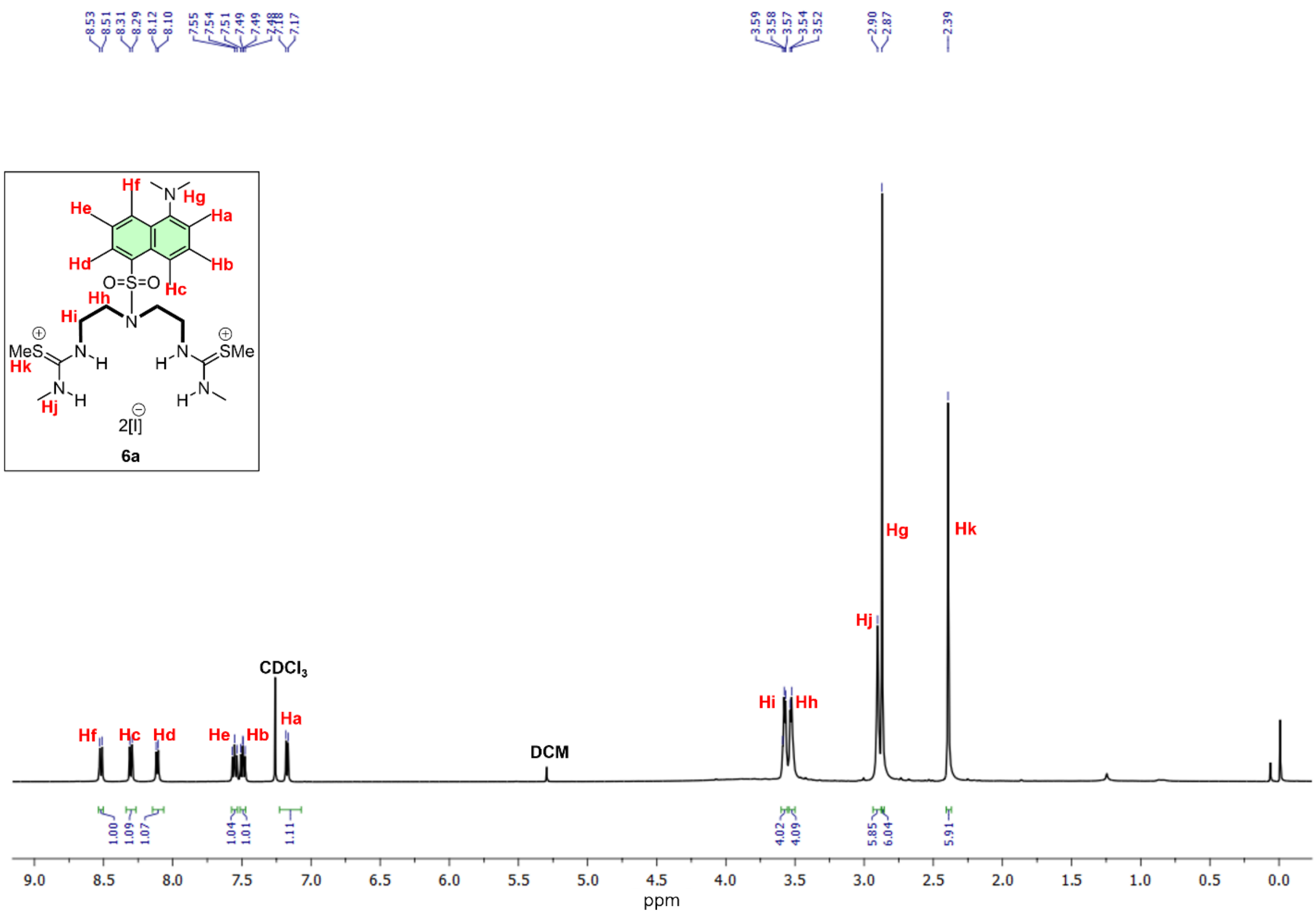


**Figure S4**: $^{1}H$ NMR (500 MHz, $CDCl_3$) of isothiouronium iodide **6a**.

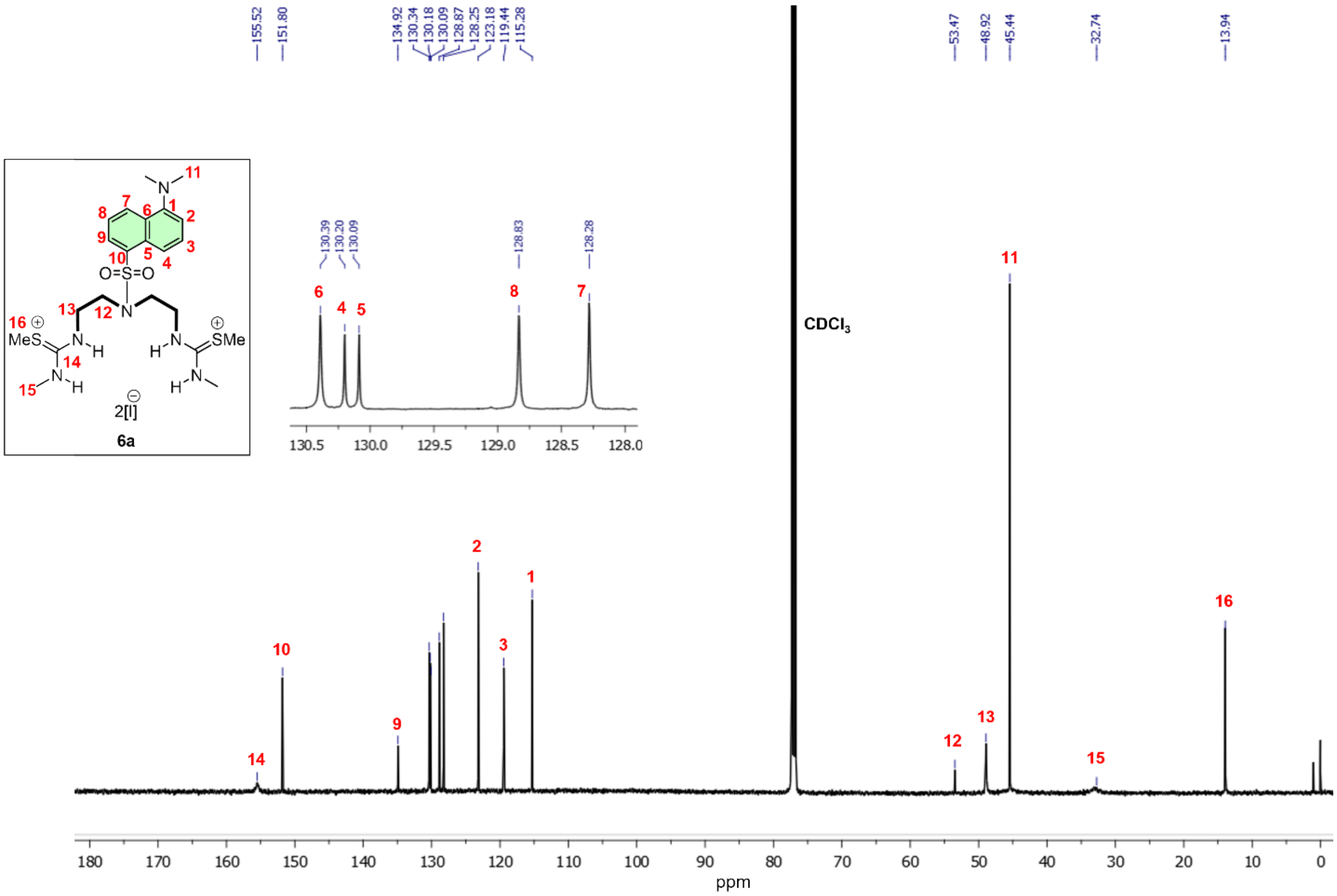

**Figure S5**: $^{13}$C NMR (125 MHz, $CDCl_3$) of isothiouronium iodide **6a**.

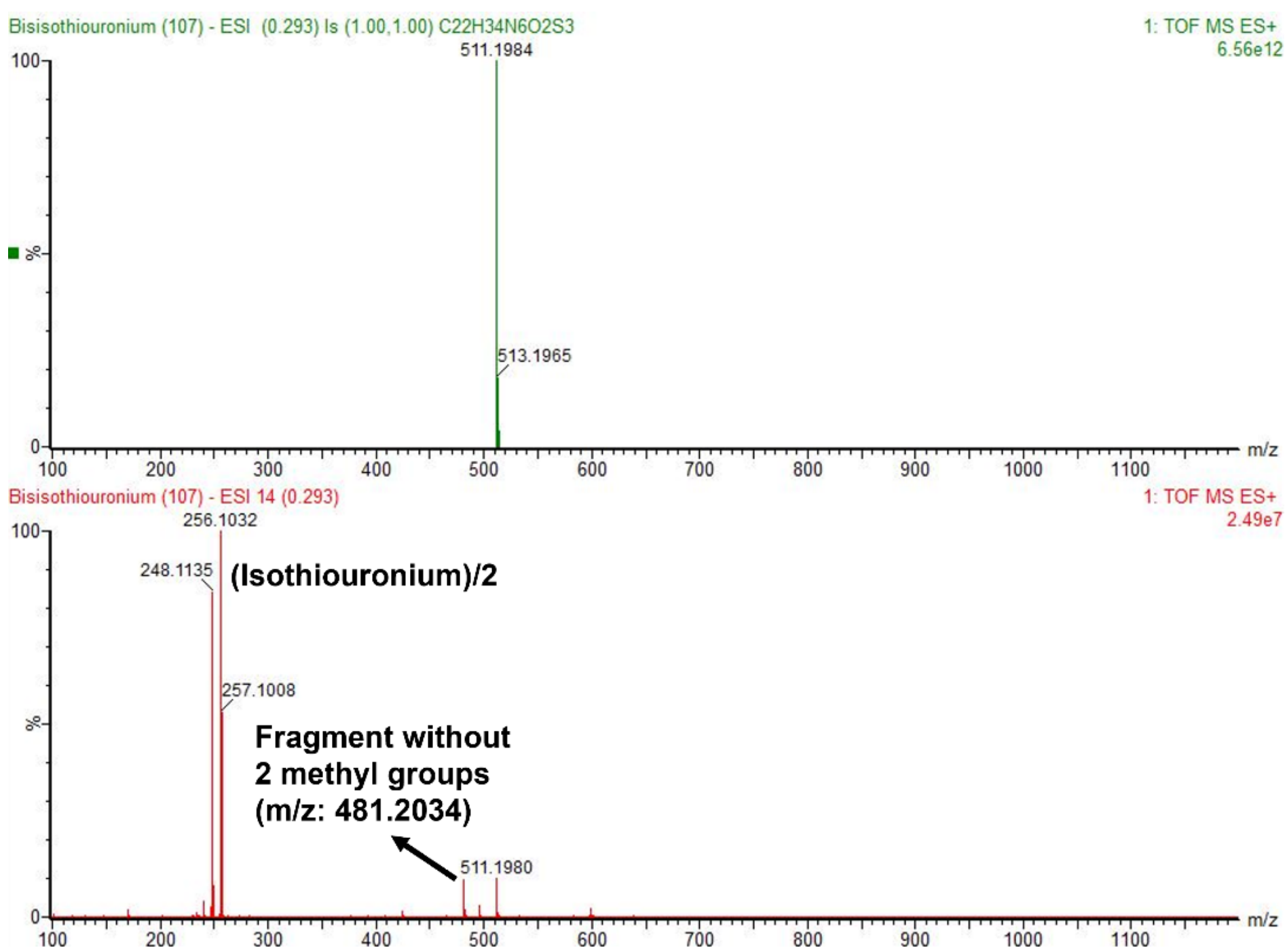


**Figure S6**: HRMS data of isothiouronium **6.** Top panel shows calculated mass and bottom panel shows observed mass.

(((((5-(dimethylamino)naphthalen-1-yl)sulfonyl)azanediyl)bis(ethane-2,1-diyl))bis(azanediyl))bis(aminomethaniminium chloride) (Guanidinium **7**): To a solution of diamine 1 (0.06 g, 0.17 mmol) in DMF (2 mL), DIPEA (0.09 mL, 0.53 mmol) and 1*H*-Pyrazole-1-carboxamidine hydrochloride (0.052 g, 0.35 mmol) was added, and the mixture was left to stir at 60 ºC overnight. Upon completion of the reaction as indicated by TLC, solvent was evaporated under reduced pressure and the crude was recrystallized from a DCM/pentane mixture to yield the pure product 7 as yellow solid (0.068 g, 78%)[4]; $^1$H NMR (500 MHz, $D_2O$) δ 8.52 (d, *J* = 8.6 Hz, 1H), 8.30 (dd, *J* = 7.5, 0.9 Hz, 1H), 7.98 (d, *J* = 8.6 Hz, 1H), 7.68 (dd, *J* = 12.2, 4.8 Hz, 2H), 7.40 (d, *J* = 7.6 Hz, 1H), 3.66 – 3.56 (m, 4H), 3.37 – 3.28 (m, 4H), 2.85 (s, 6H); $^{13}$C NMR (125 MHz, $D_2O$) δ 156.24, 150.55, 132.20, 131.46, 130.91, 129.32, 129.04, 128.97, 123.94, 119.14, 116.13, 44.88, 43.37, 37.45; HRMS (ESI) m/z: calcd. for $C_{18}H_{30}N_8O_2S$ [M-H] 421.2134 found 421.2151 (□□m□= 4.0 ppm) and [M/2] 211.1106 found 211.1096 (□□m□= 4.7 ppm)

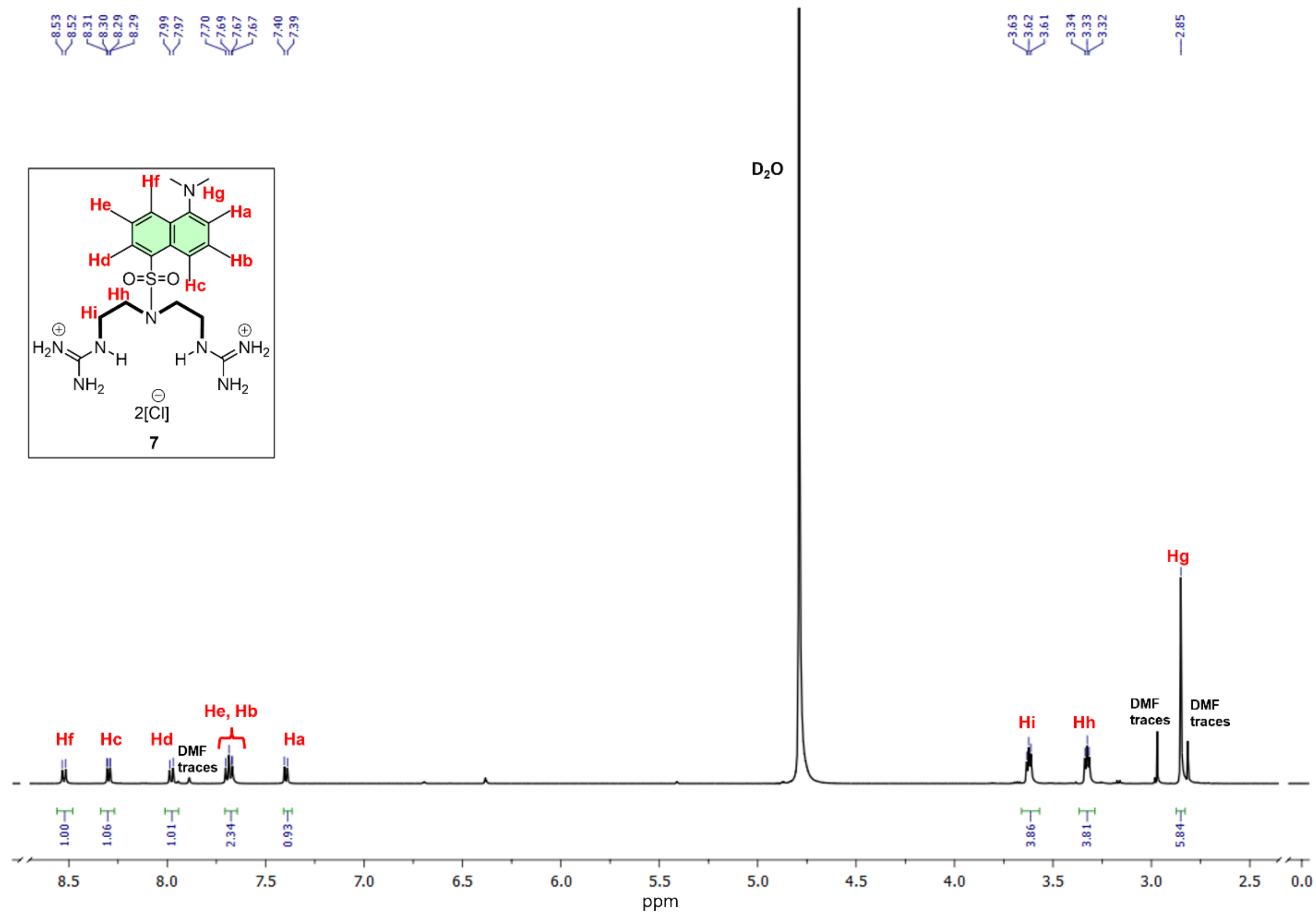


**Figure S7**: $^1$H NMR (500 MHz, $D_2O$) of guanidinium **7**.

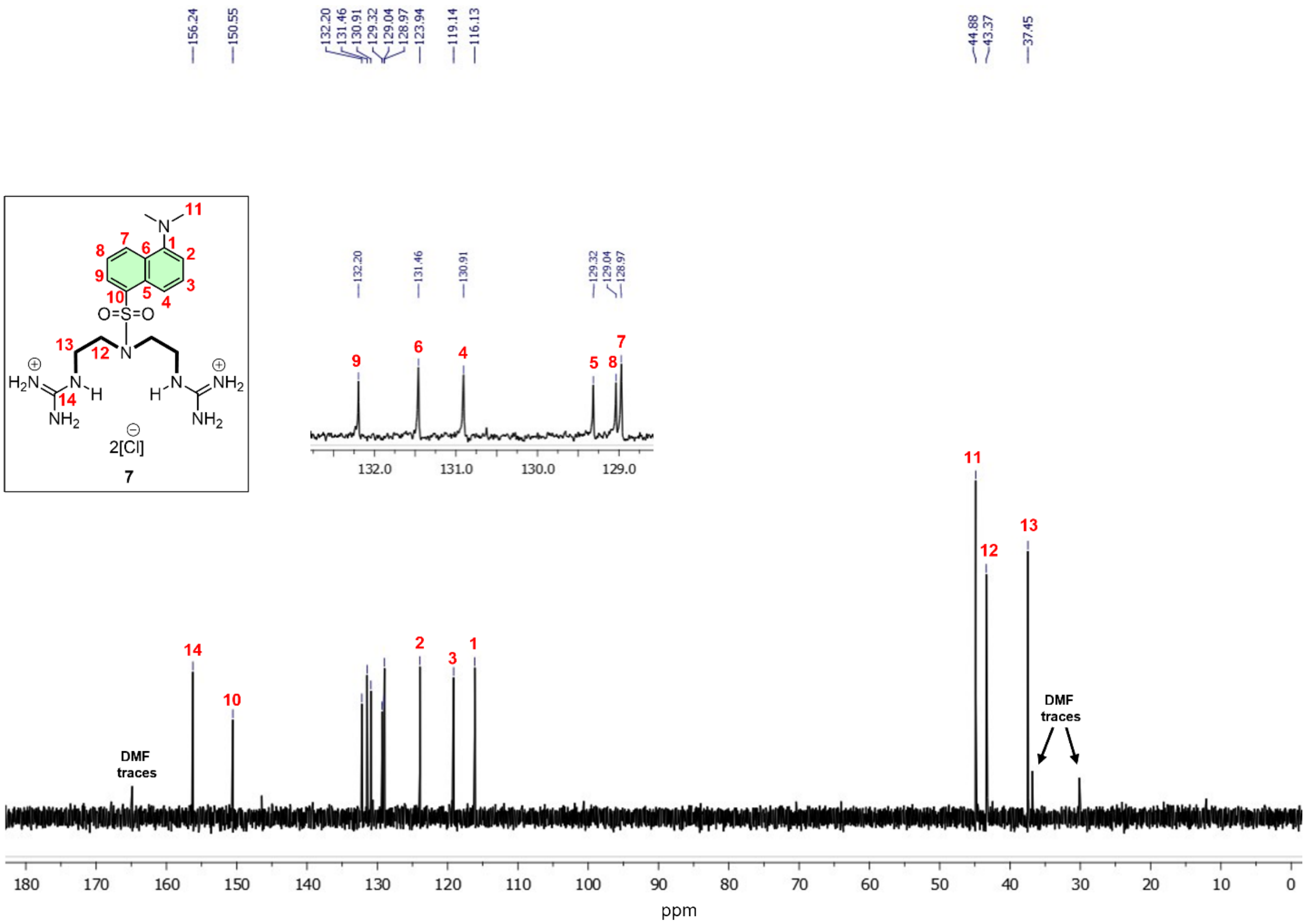


**Figure S8**: $^{13}C$ NMR (125 MHz, $D_2O$) of guanidinium **7**.

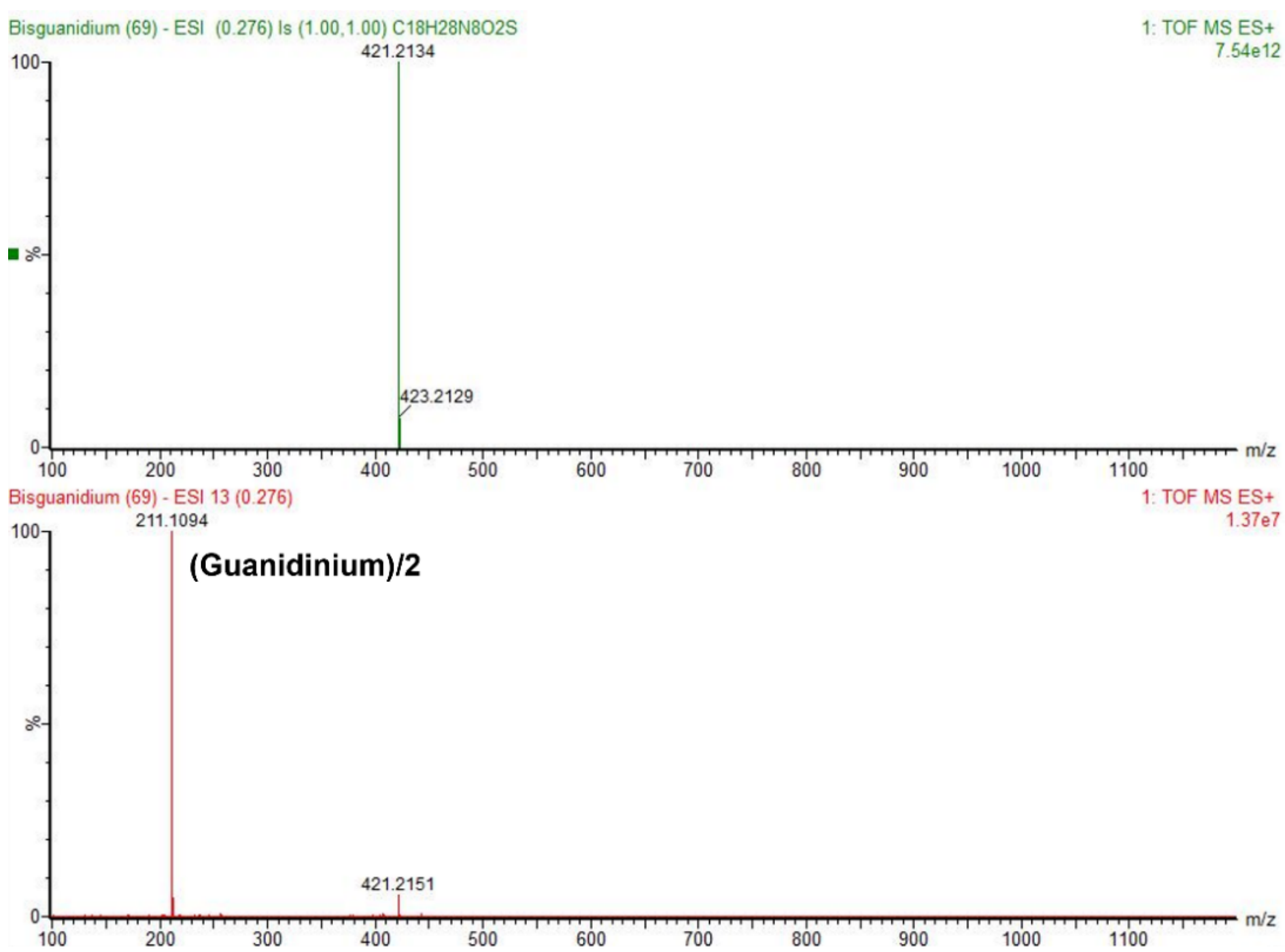


**Figure S9**: HRMS data of guanidinium **7**. Top panel shows calculated mass and bottom panel shows observed mass.

## $^1$H NMR Titration Experiments

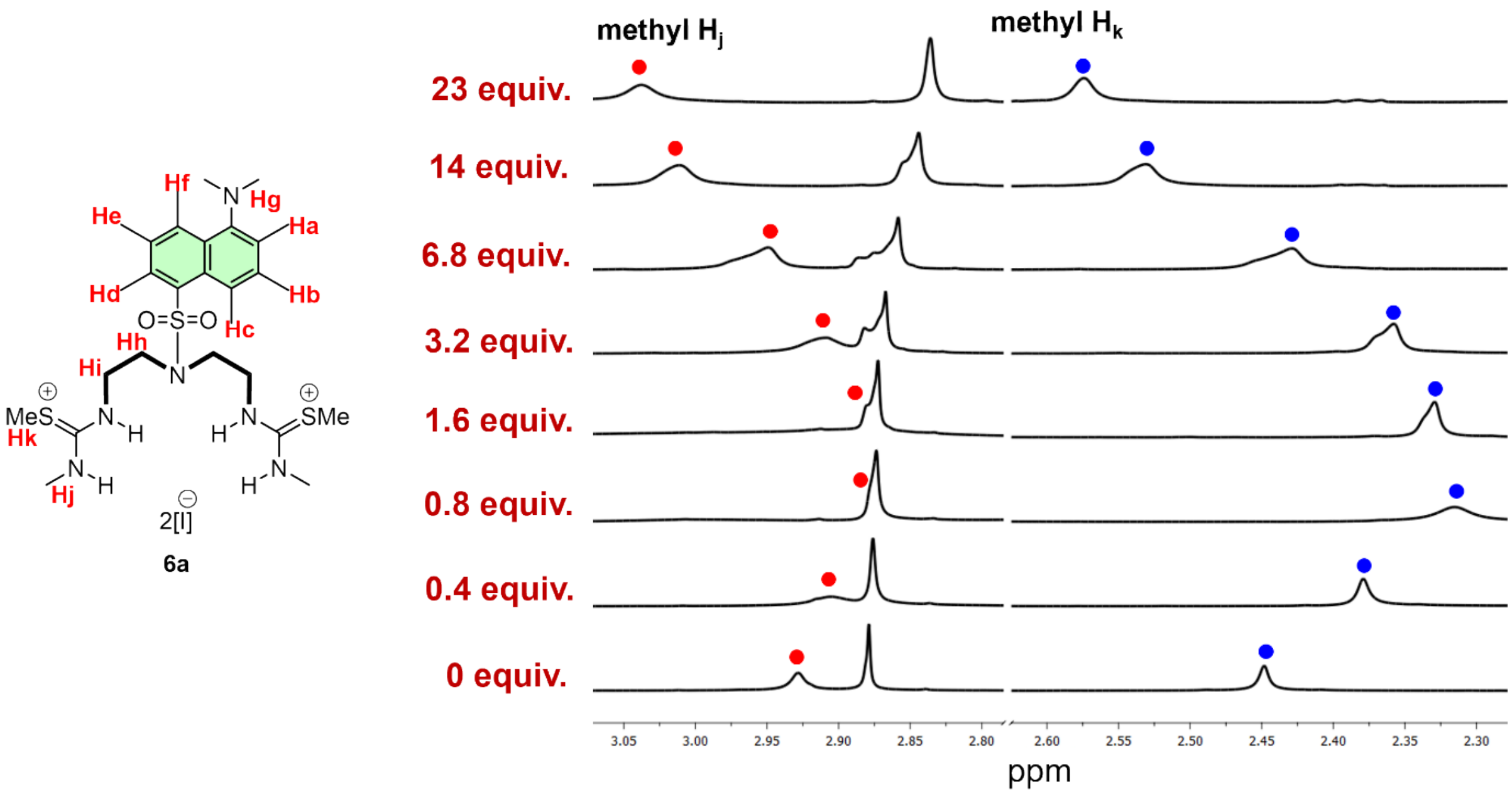


**Figure S10**: $^1$H NMR (500 MHz, $CDCl_3$) titration of isothiouronium **6a** (4.6 mM) with $TBAH_2PO_4$. Methyl protons $H_j$ (●) and $H_k$ (●) show downfield chemical shift at low concentrations of Pi and, reverses at higher concentration of Pi suggesting the possibility of two distinct binding processes which were later confirmed by fluorescence titration experiments.

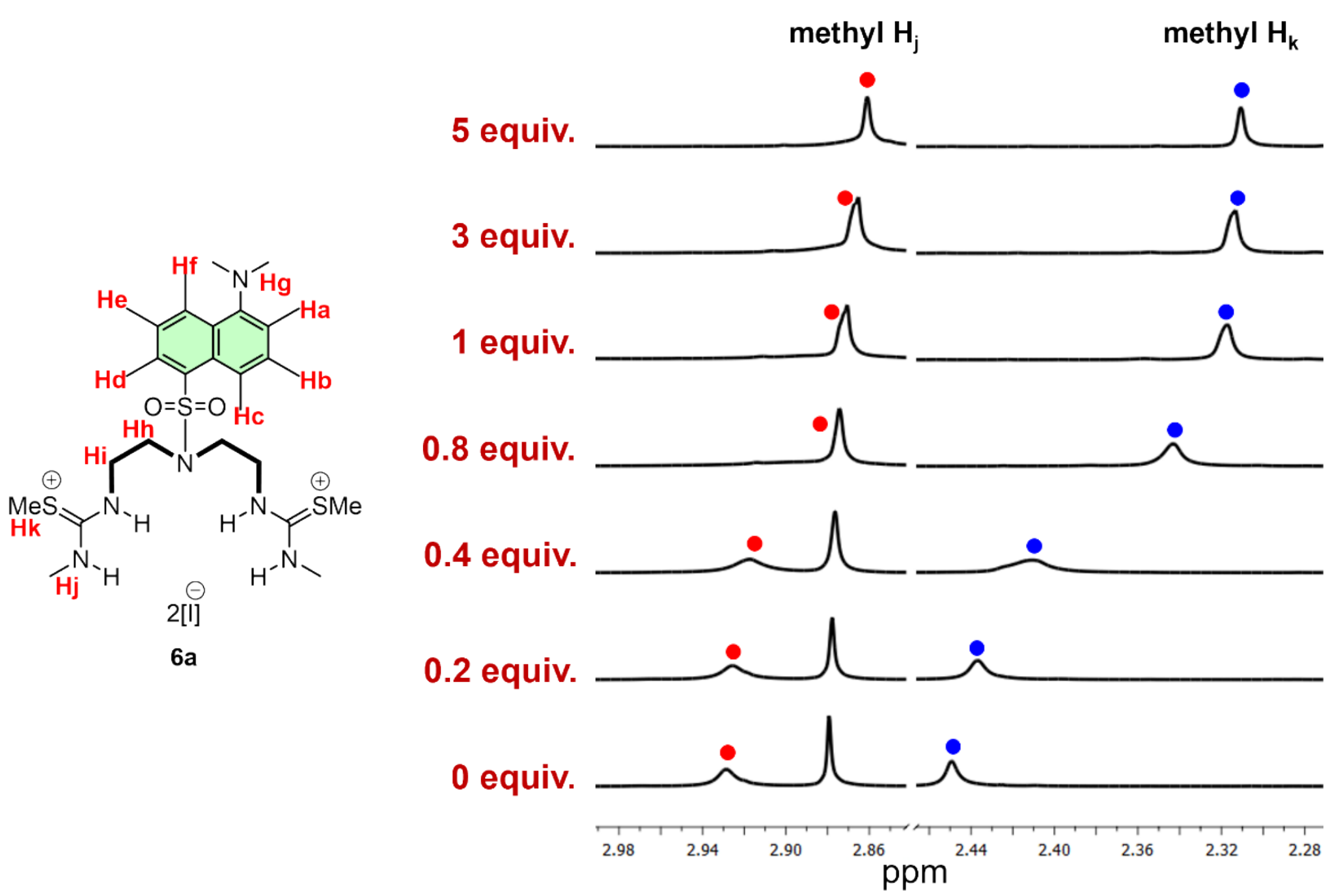


Figure S11: $^1$H NMR (500 MHz, $CDCl_3$) titration of isothiouronium 6a (4.6 mM) with $(TBA)_3HP_2O_7$. Methyl protons $H_j$ (●) and $H_k$ (●) show downfield chemical shift upon complexation with PPi, and this unidirectional shift suggests single binding event, and this was confirmed by fluorescence titration experiments.

## UV-Vis and High-Throughput Titration Experiments

### (a) Diamine 1

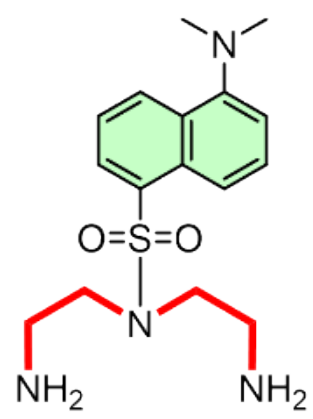


**1**

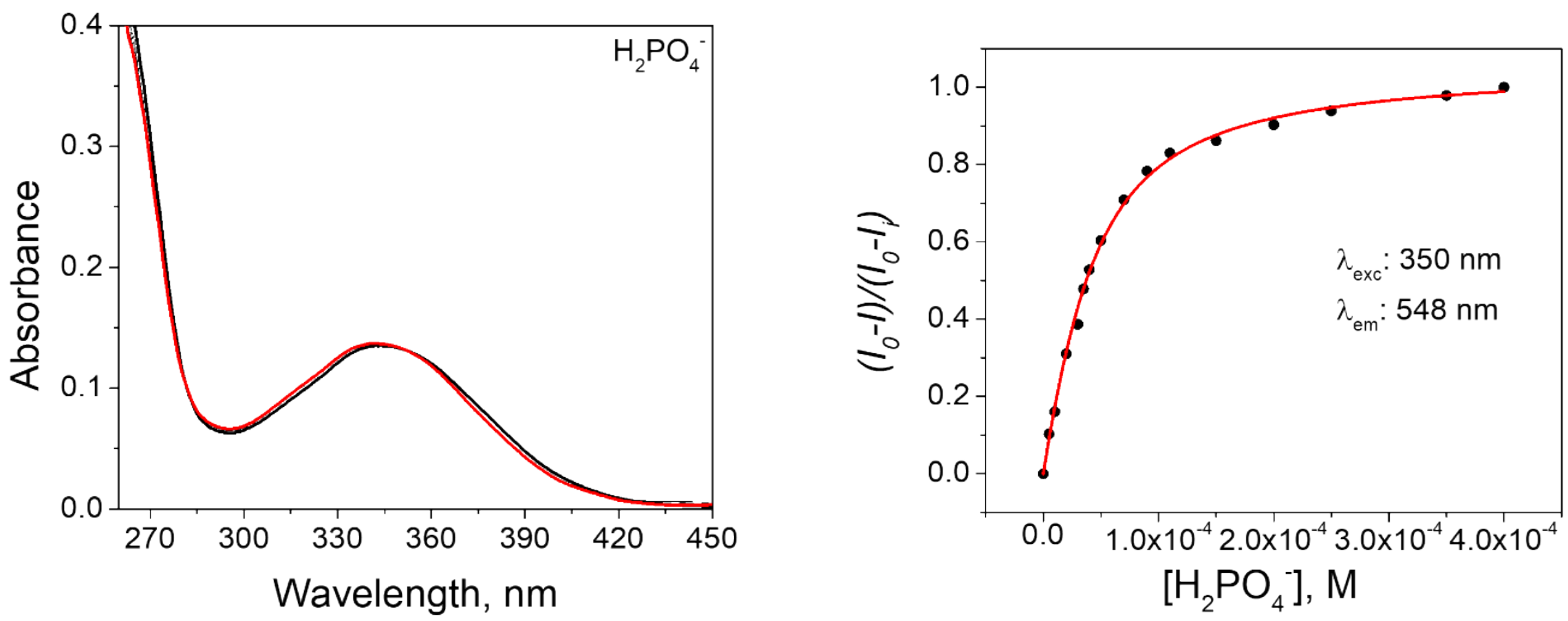


**Figure S12**: UV-Vis absorption spectrum (30 μM of 1) and isotherm corresponding to change in fluorescence intensity as an average of 20 replicas obtained from the microplate reader monitored at $\lambda_{em}$ = 548 nm for 15 μM of 1 in 10% water in DMSO (pH 5.5) using $\lambda_{exc}$ = 350 nm, upon incremental addition of Pi.

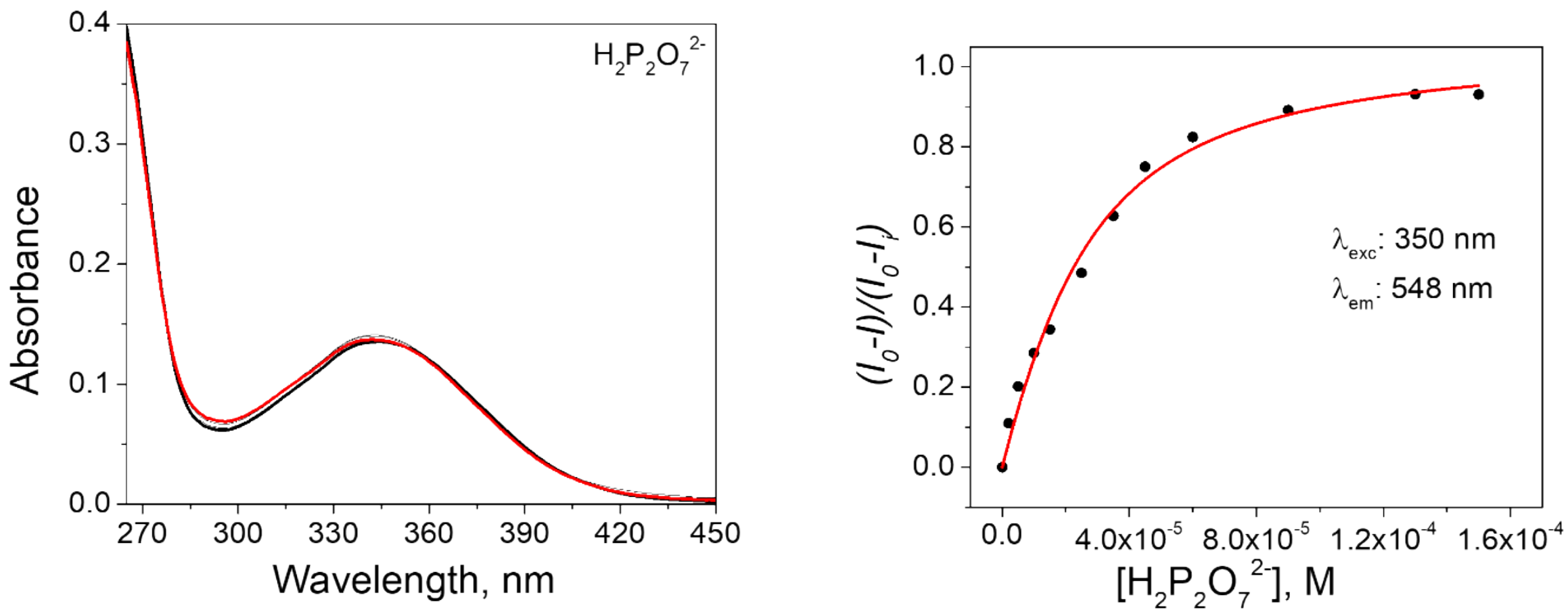


**Figure S13:** UV-Vis absorption spectrum (30 μM of 1) and isotherm corresponding to change in fluorescence intensity as an average of 20 replicas obtained from the microplate reader monitored at $\lambda_{em}$ = 548 nm for 15 μM of 1 in 10% water in DMSO (pH 5.5) using $\lambda_{exc}$ = 350 nm, upon incremental addition of PPi.

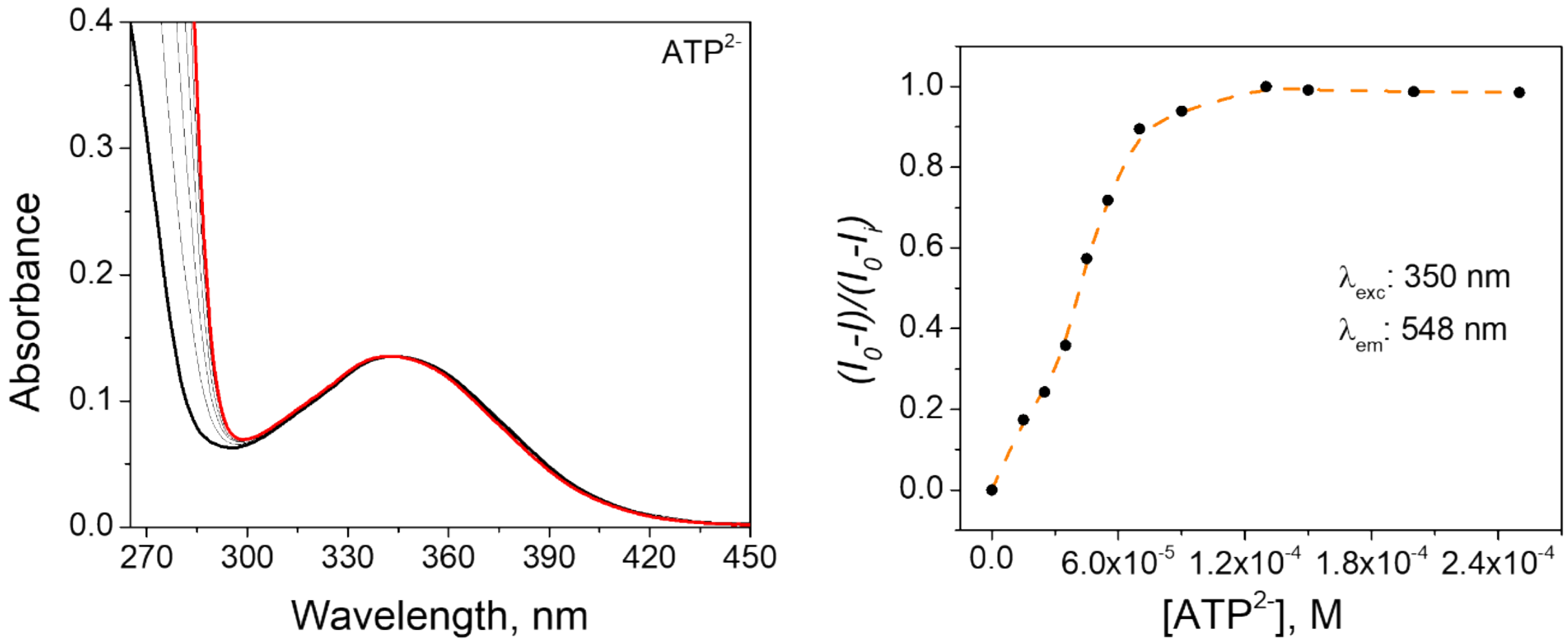


**Figure S14:** UV-Vis absorption spectrum (30 μM of **1**) and isotherm corresponding to change in fluorescence intensity as an average of 20 replicas obtained from the microplate reader monitored at $\lambda_{em}$ = 548 nm for 15 μM of **1** in 10% water in DMSO (pH 5.5) using $\lambda_{exc}$ = 350 nm, upon incremental addition of ATP.

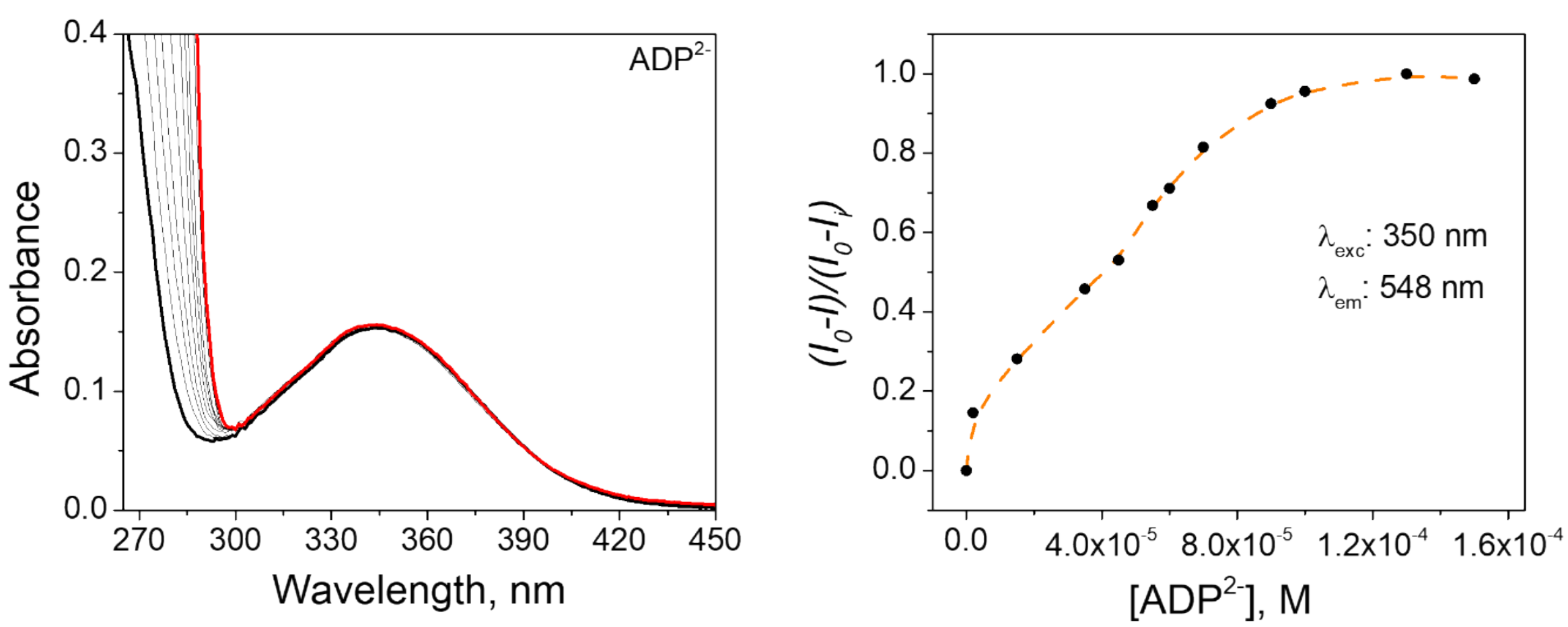


**Figure S15**: UV-Vis absorption spectrum (30 μM of **1**) and isotherm corresponding to change in fluorescence intensity as an average of 20 replicas obtained from the microplate reader monitored at $\lambda_{em}$ = 548 nm for 15 μM of **1** in 10% water in DMSO (pH 5.5) using $\lambda_{exc}$ = 350 nm, upon incremental addition of ADP.

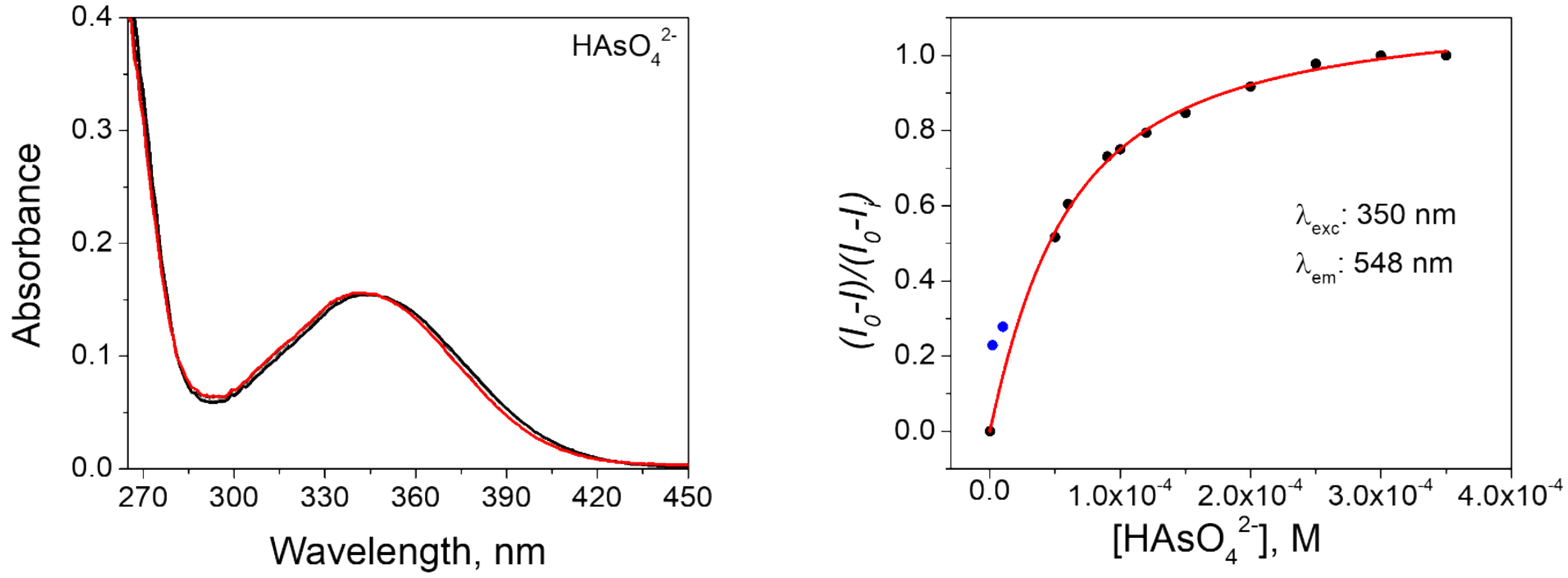


**Figure S16**: UV-Vis absorption spectrum (30 $\mu$M of **1**) and isotherm corresponding to change in fluorescence intensity as an average of 20 replicas obtained from the microplate reader monitored at $\lambda_{em}$ = 548 nm for 15 $\mu$M of **1** in 10% water in DMSO (pH 5.5) using $\lambda_{exc}$ = 350 nm, upon incremental addition of Arsenate.

## (b) Isothiouronium 6

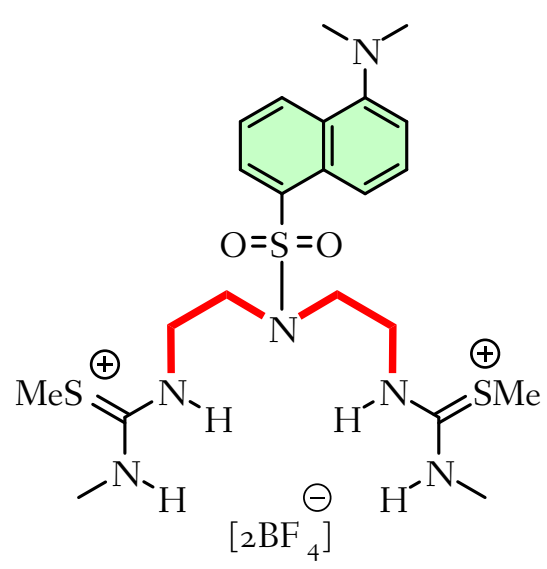

6

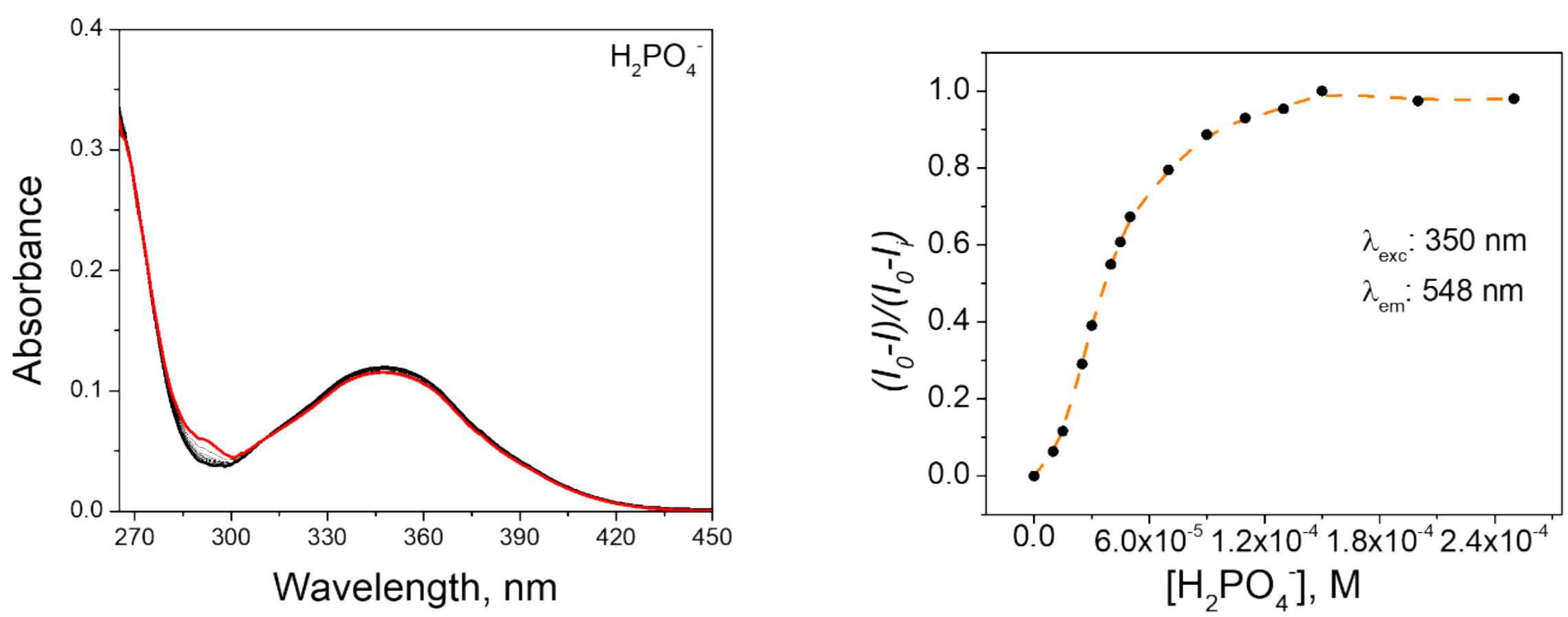


**Figure S17:** UV-Vis absorption spectrum (30 $\mu$M of **6**) and isotherm corresponding to change in fluorescence intensity as an average of 20 replicas obtained from the microplate reader monitored at $\lambda_{em}$ = 548 nm for 15 $\mu$M of **6** in 10% water in DMSO (pH 5.5) using $\lambda_{exc}$ = 350 nm, upon incremental addition of Pi.

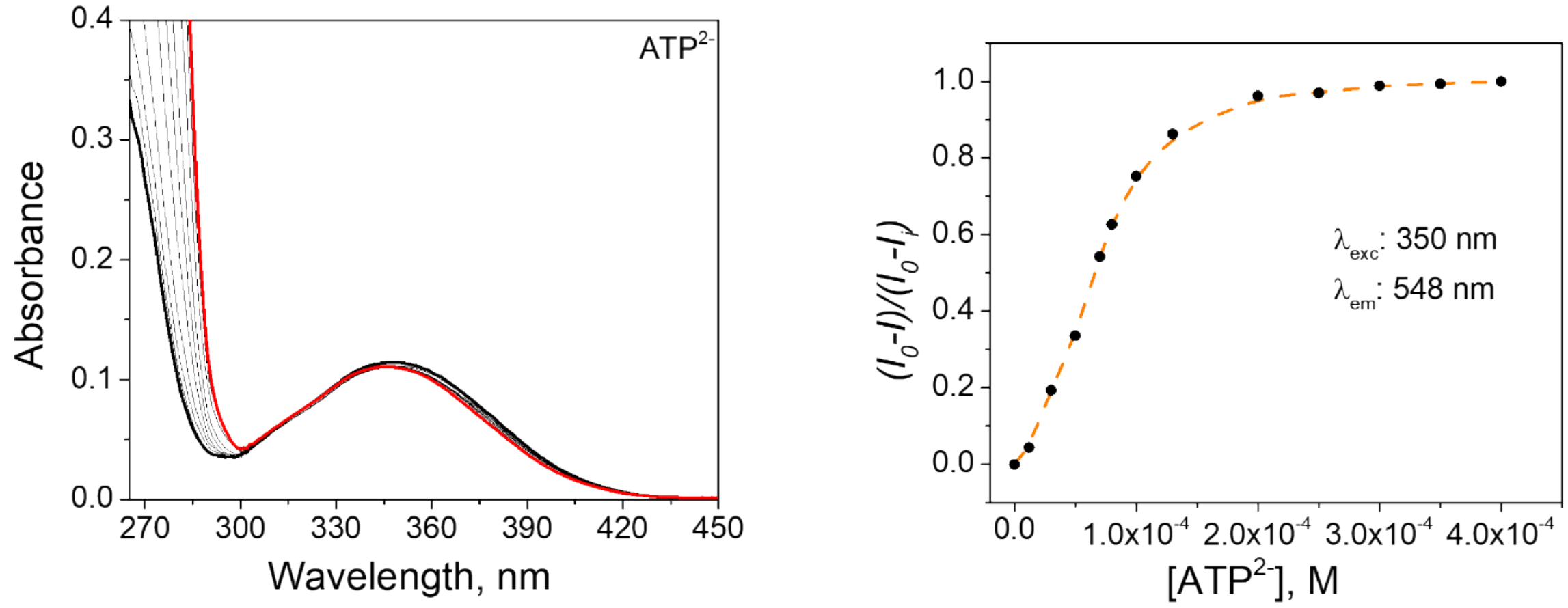


**Figure S18**: UV-Vis absorption spectrum (30 $\mu$M of **6**) and isotherm corresponding to change in fluorescence intensity as an average of 20 replicas obtained from the microplate reader monitored at λ$_{em}$ = 548 nm for 15 $\mu$M of **6** in 10% water in DMSO (pH 5.5) using λ$_{exc}$ = 350 nm, upon incremental addition of ATP.

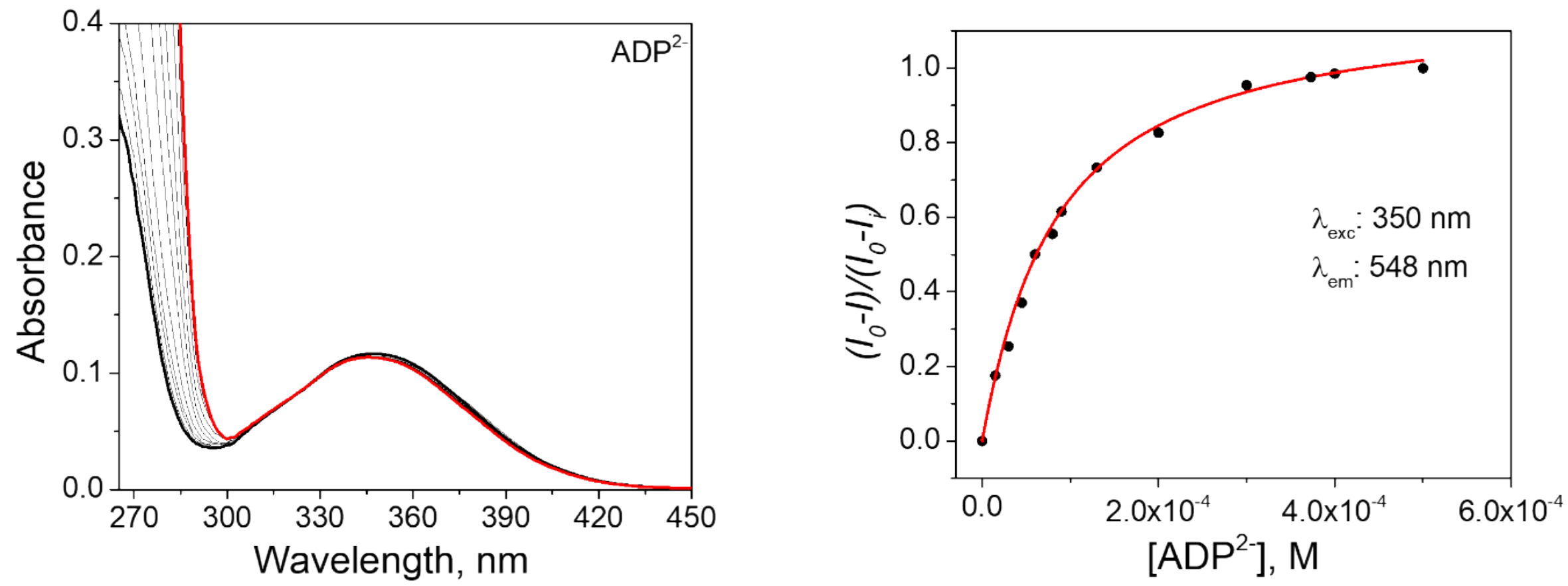


**Figure S19**: UV-Vis absorption spectrum (30 $\mu$M of **6**) and isotherm corresponding to change in fluorescence intensity as an average of 20 replicas obtained from the microplate reader monitored at λ$_{em}$ = 548 nm for 15 $\mu$M of **6** in 10% water in DMSO (pH 5.5) using λ$_{exc}$ = 350 nm, upon incremental addition of ADP.

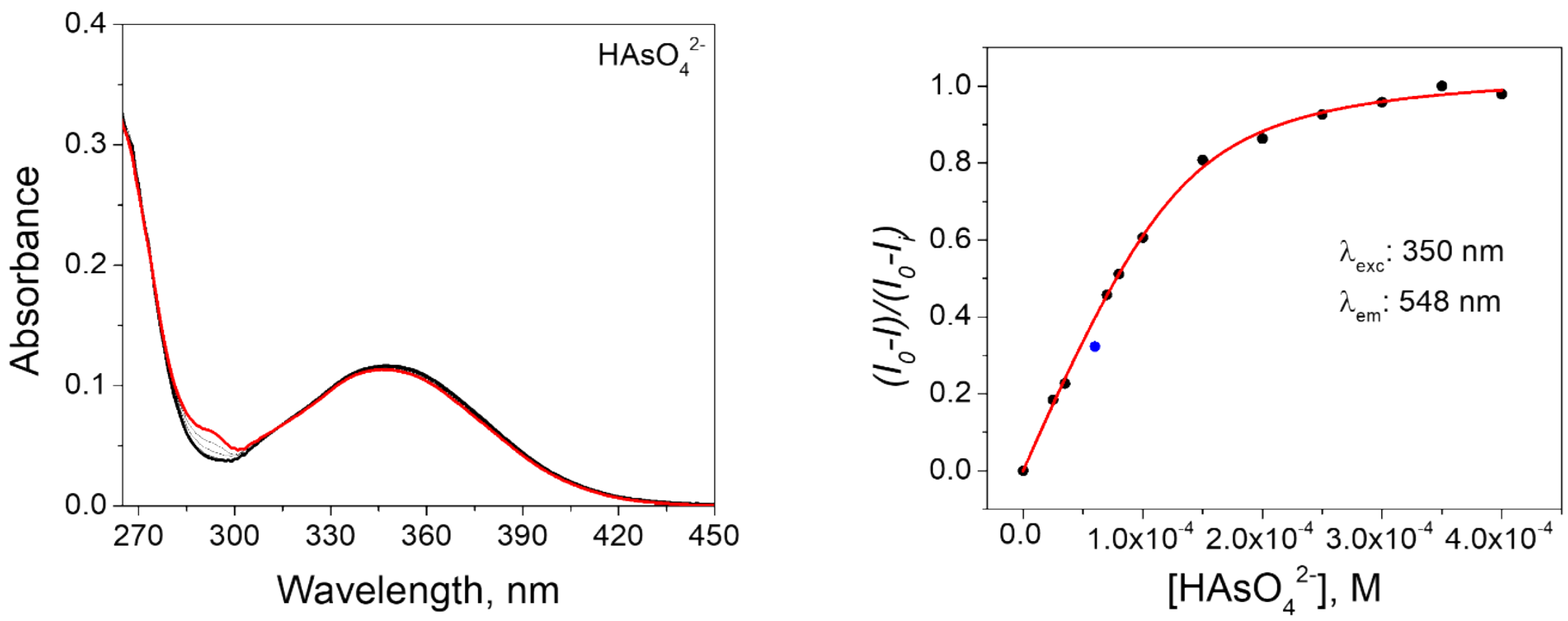


**Figure S20**: UV-Vis absorption spectrum (30 $\mu$M of **6**) and isotherm corresponding to change in fluorescence intensity as an average of 20 replicas obtained from the microplate reader monitored at $\lambda_{em}$ = 548 nm for 15 $\mu$M of **6** in 10% water in DMSO (pH 5.5) using $\lambda_{exc}$ = 350 nm, upon incremental addition of Arsenate.

## UV-Vis and Fluoresence Titration Experiments

# Diamine 1

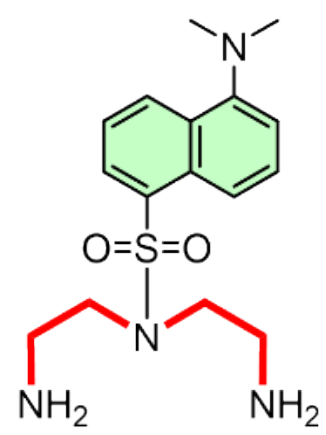


**1**

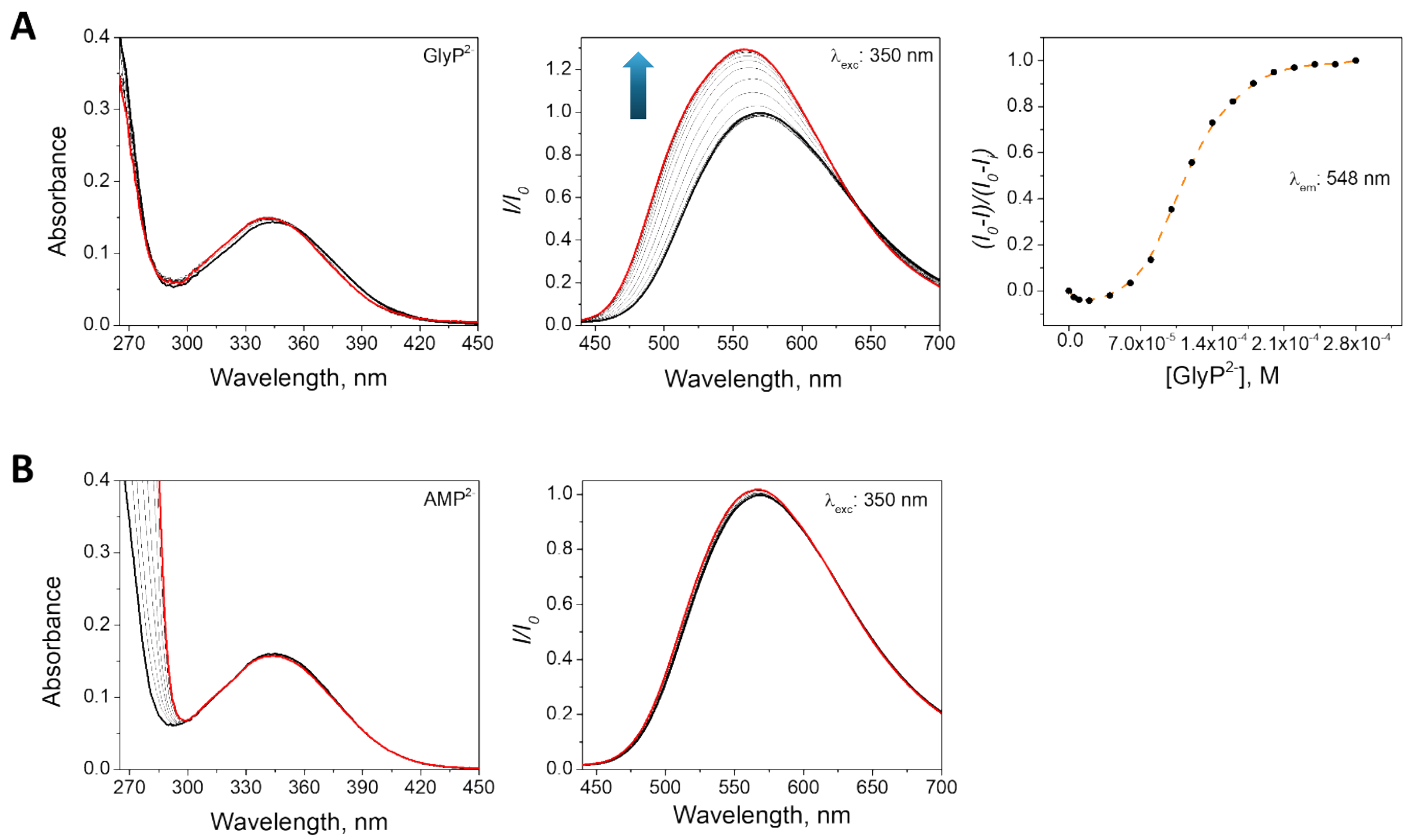


**Figure S21**: **A**. Left) UV-Vis titration (30 $\mu$M of **1**), center) fluorescence titration ($\lambda_{exc}$ = 350 nm), and right) binding isotherm of fluorescence titration at $\lambda_{em}$ = 548 nm of **1** (15 $\mu$M) in 10% water in DMSO at pH 5.5, upon incremental addition of GlyP. **B**. UV-Vis titration and fluorescence titration at $\lambda_{exc}$ = 350 nm of **1** (15 $\mu$M) in 10% water in DMSO at pH 5.5, upon incremental addition of AMP.

## Isothiouronium 6

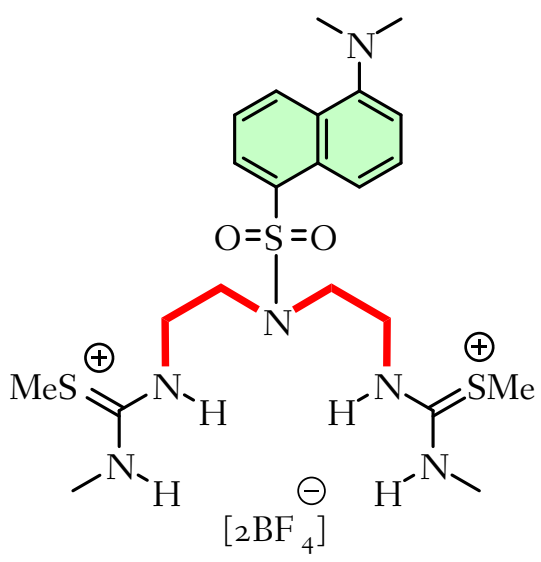


6

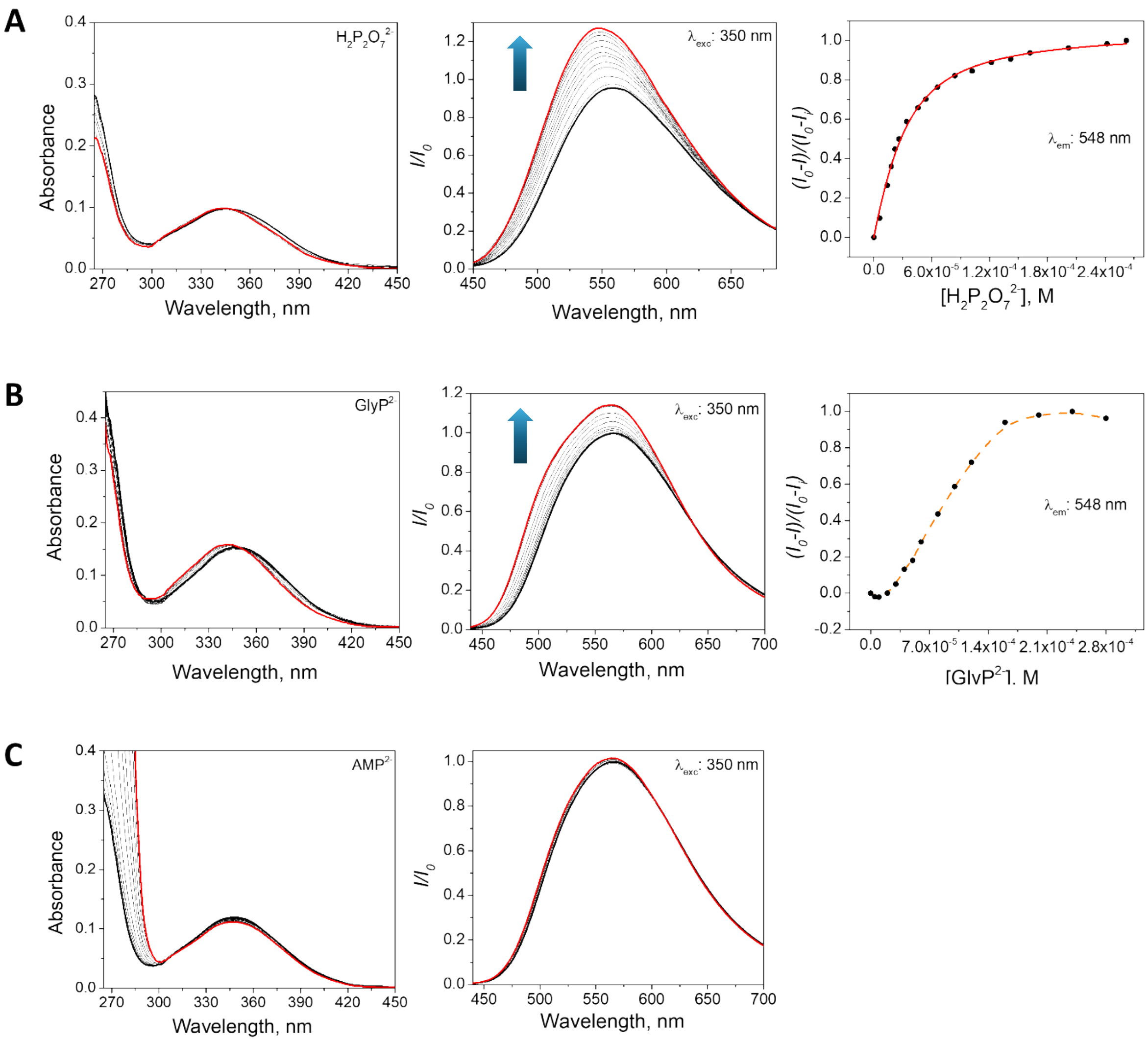


**Figure S22**: **A.** Left) UV-Vis titration (30 $\mu$M of **6**), center) fluorescence titration (λ$_{exc}$ = 350 nm), and right) binding isotherm of fluorescence titration at λ$_{em}$ = 548 nm of **6** (15 $\mu$M) in 10% water in DMSO at pH 5.5, upon incremental addition of PPi. **B.** Left) UV-Vis titration (30 $\mu$M), center) fluorescence titration (λ$_{exc}$ = 350 nm), and right) binding isotherm of fluorescence titration at λ$_{em}$ = 548 nm of **6** (15 $\mu$M) in 10% water in DMSO at pH 5.5, upon incremental addition of GlyP. **C**. UV-Vis titration and fluorescence titration at λ$_{exc}$ = 350 nm of **6** (15 $\mu$M) in 10% water in DMSO at pH 5.5, upon incremental addition of AMP.

## Guanidinium 7

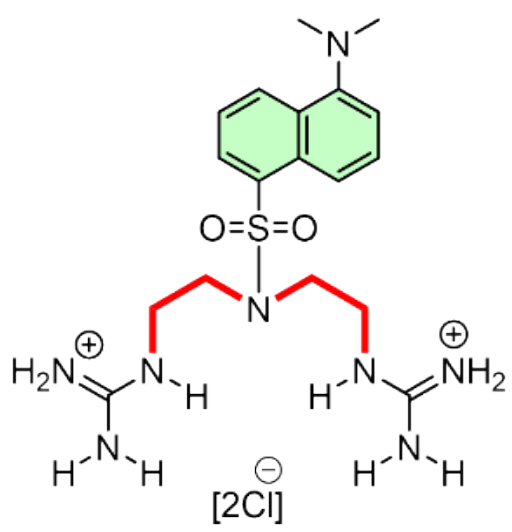


7

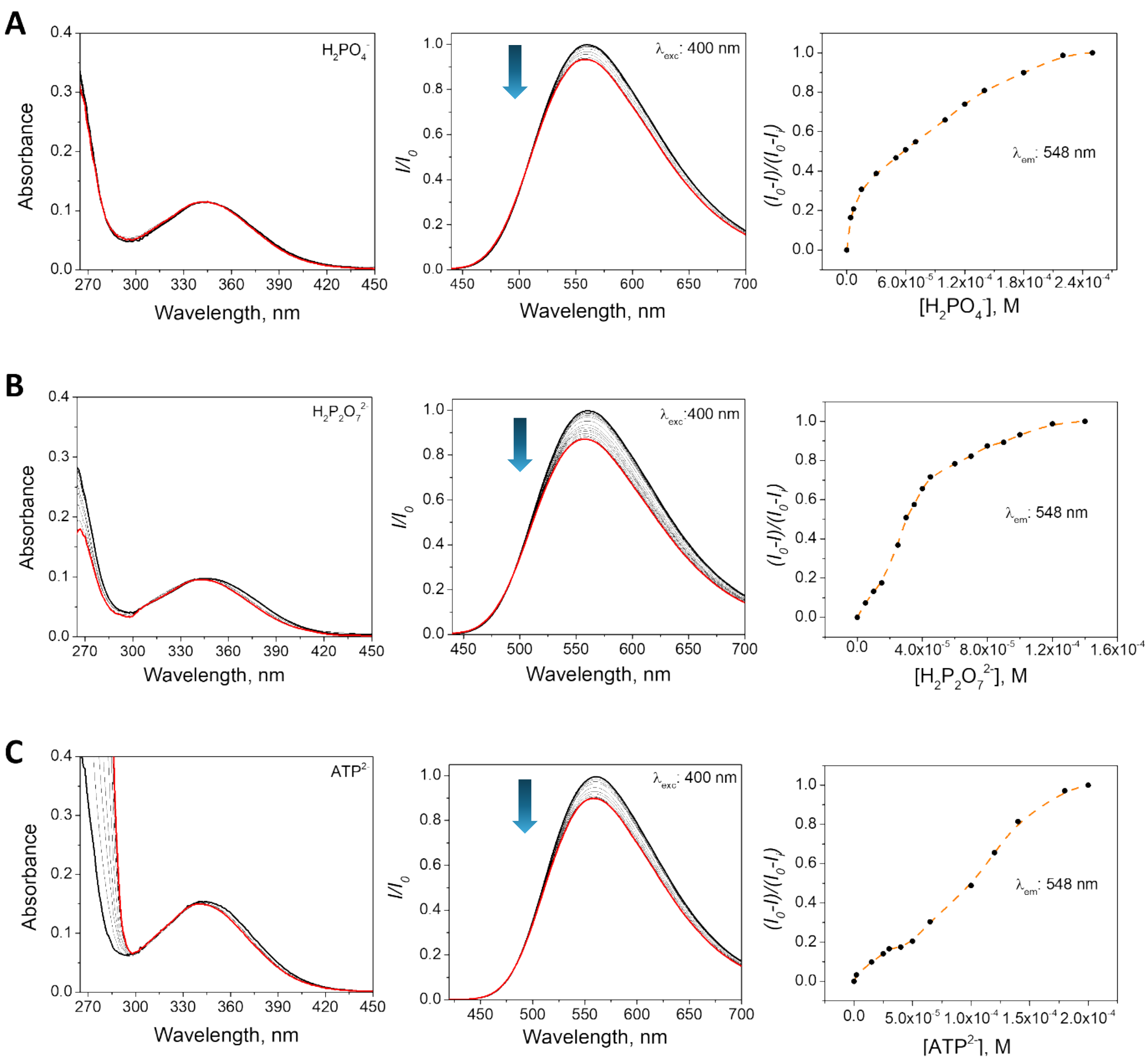

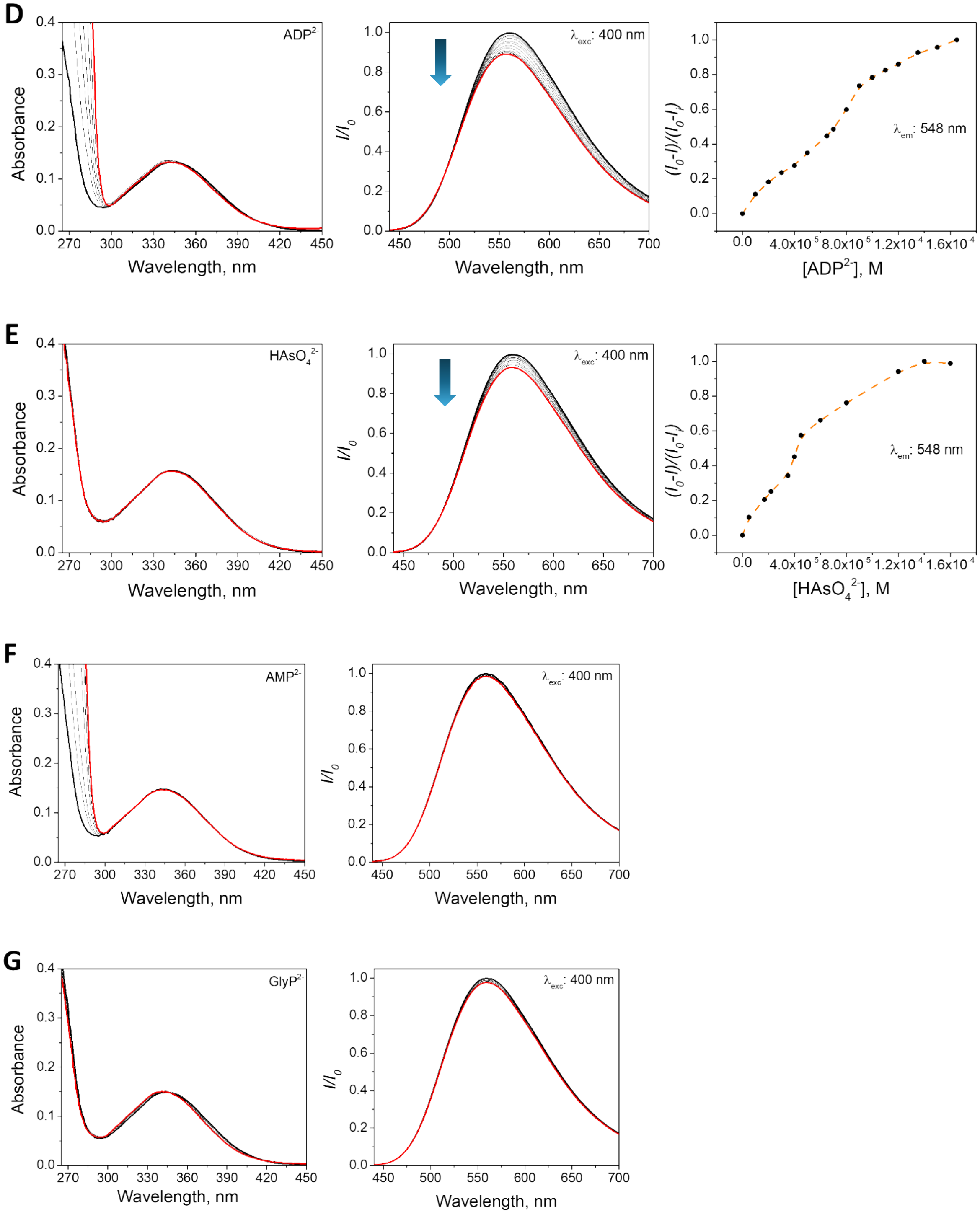


**Figure S23**: Left) UV-Vis titration (30 $\mu$M of **7**), center) fluorescence titration ($\square_{exc}$ = 350 nm), and right) binding isotherm of fluorescence titration at $\square_{em}$ = 548 nm of **7** (15 $\mu$M) in 10% water in DMSO at pH 5.5, upon incremental addition of (**A**) Pi, (**B**) PPi, (**C**) ATP, (**D**) ADP, (**E**) Arsenate, (**F**) AMP and (**G**) GlyP.

## Thiourea 5

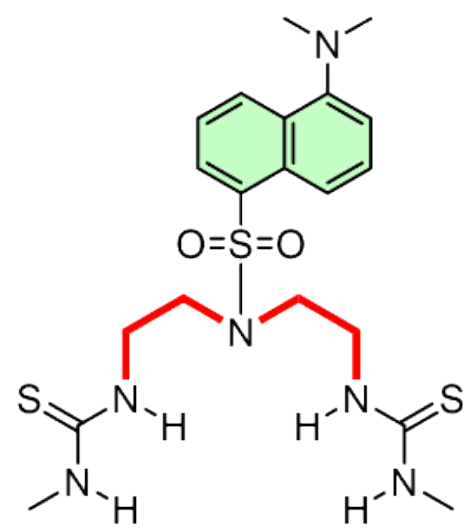


**5**

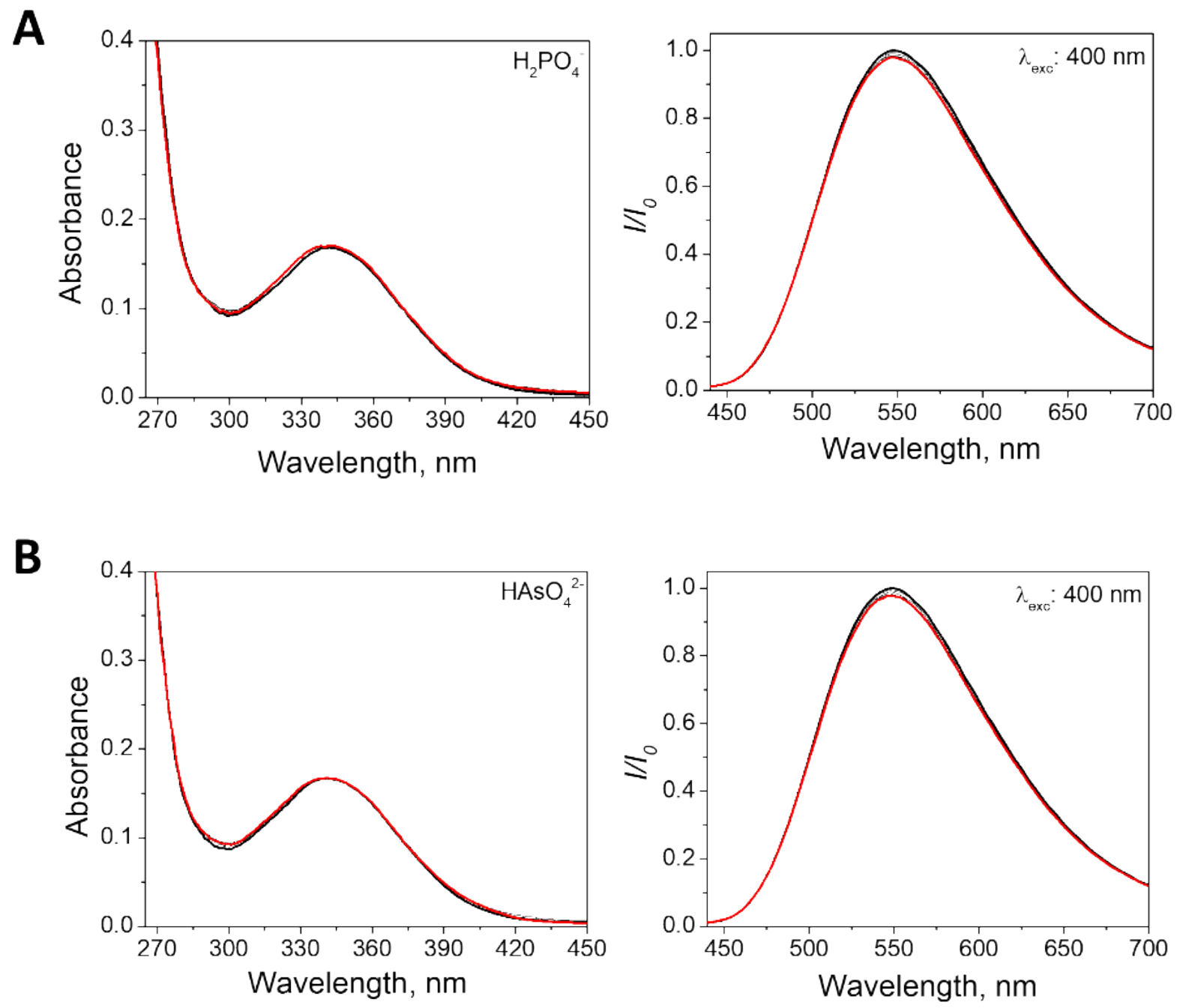


**Figure S24**: UV-Vis titration and fluorescence titration at $\square_{exc}$ = 350 nm of **5** (15 $\mu$M) in DMSO, upon incremental addition of (**A**) Pi and (**B**) Arsenate.

## Qualitative Linear Discriminant Analysis

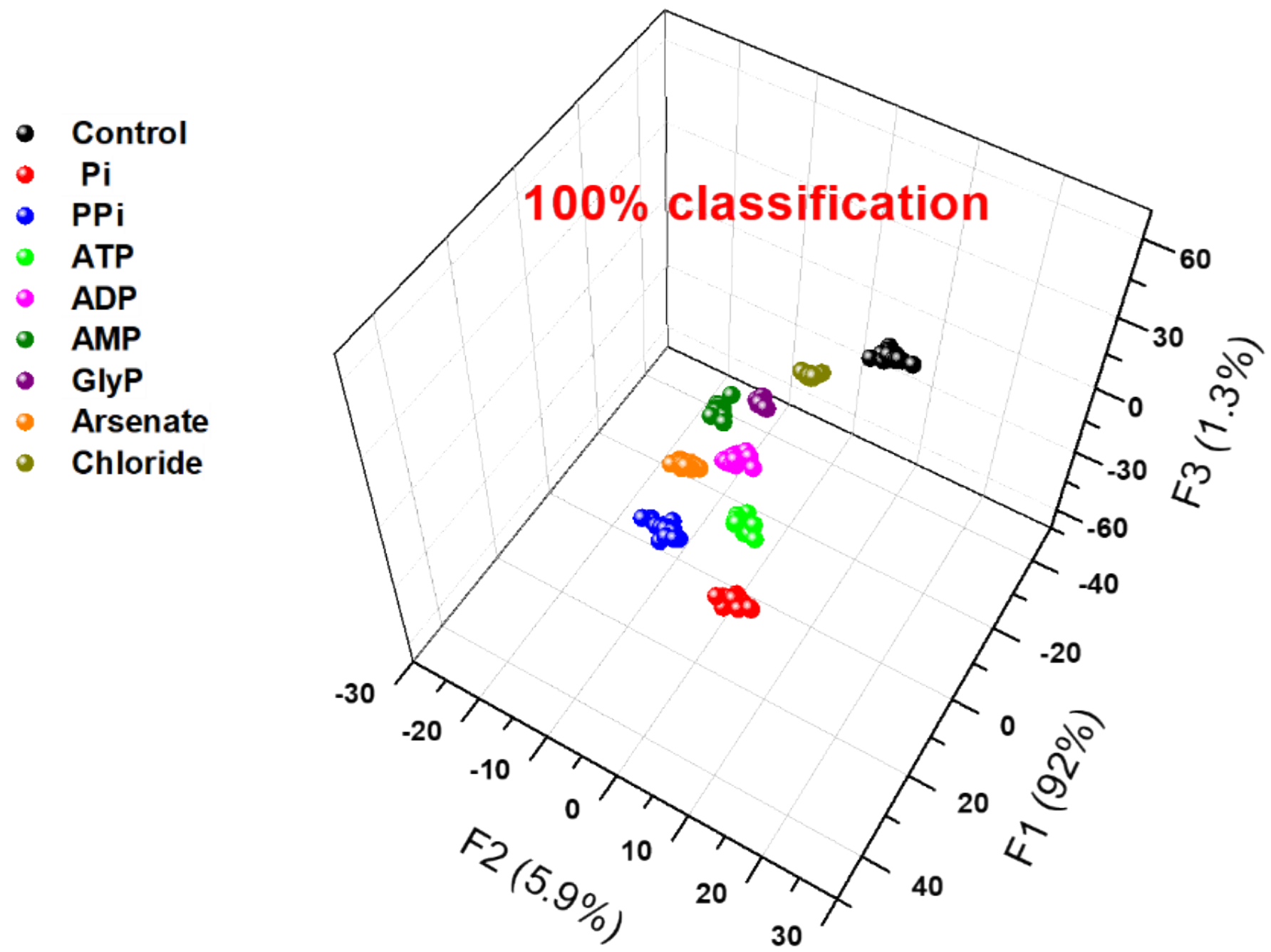


**Figure S25**: 3D graphical output of qualitative LDA displaying clusters of 8 analytes and 1 control from an array of sensors **1** and **6**. Sensors **1** and **6** (both 15 $\mu$M), each in 20 replicas (total of 180 datapoints), and a correct assignment to all classes with 100% correct classification. Measured in water-DMSO (1:9 v/v, pH 5.5) upon addition of phosphates as sodium salts in water (3 equiv.).

**A**

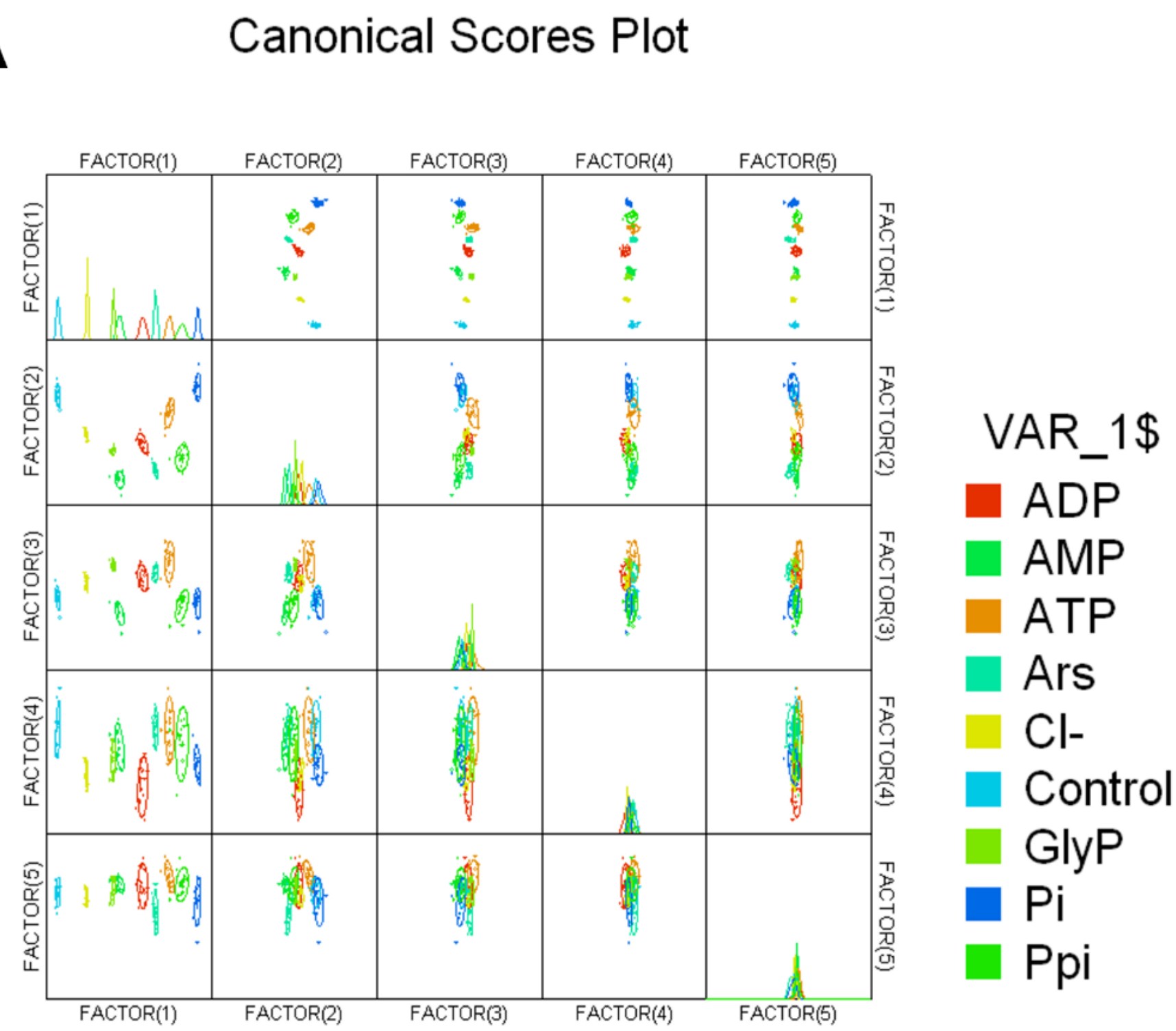


**B**

Jackknifed Classification Matrix

| | ADP | AMP | ATP | Ars | Cl- | Control | GlyP | Pi | Ppi | %correct |
|---|---|---|---|---|---|---|---|---|---|---|
| ADP | 20 | 0 | 0 | 0 | 0 | 0 | 0 | 0 | 0 | 100 |
| AMP | 0 | 20 | 0 | 0 | 0 | 0 | 0 | 0 | 0 | 100 |
| ATP | 0 | 0 | 20 | 0 | 0 | 0 | 0 | 0 | 0 | 100 |
| Ars | 0 | 0 | 0 | 20 | 0 | 0 | 0 | 0 | 0 | 100 |
| Cl- | 0 | 0 | 0 | 0 | 20 | 0 | 0 | 0 | 0 | 100 |
| Control | 0 | 0 | 0 | 0 | 0 | 20 | 0 | 0 | 0 | 100 |
| GlyP | 0 | 0 | 0 | 0 | 0 | 0 | 20 | 0 | 0 | 100 |
| Pi | 0 | 0 | 0 | 0 | 0 | 0 | 0 | 20 | 0 | 100 |
| Ppi | 0 | 0 | 0 | 0 | 0 | 0 | 0 | 0 | 19 | 100 |
| Total | 20 | 20 | 20 | 20 | 20 | 20 | 20 | 20 | 19 | 100 |

**Figure S26**: LDA canonical scores plot (A) and Jackknifed Classification matrix (B) for array of sensors **1** and **6**, and its complexes with eight anions of interest (50 $\mu$M) along with a control (water).

## Quantitative Linear Discriminant Analysis

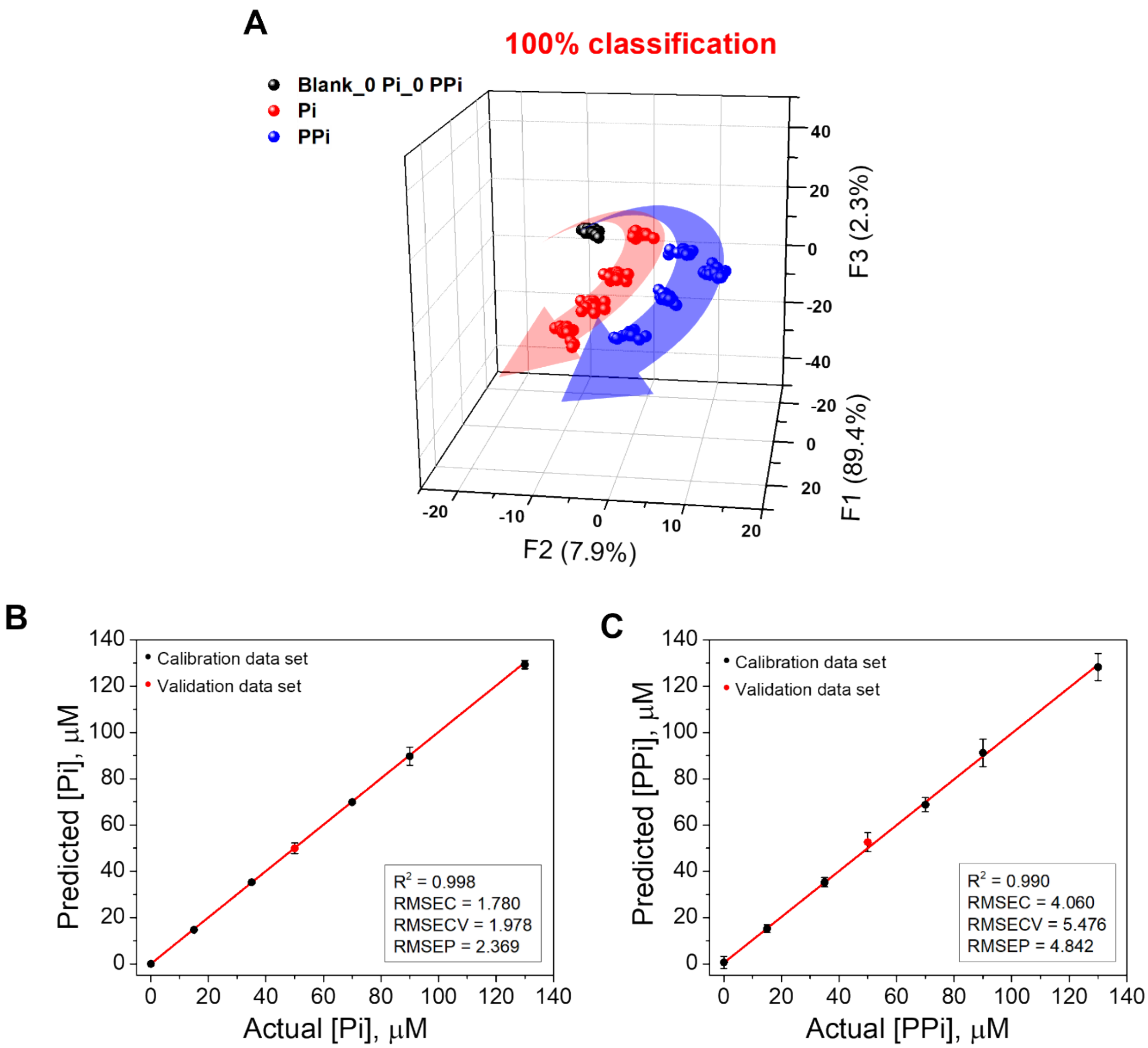


**Figure S27**: Quantitative analysis of Pi and PPi from array of sensors **1** and **6**. (Panel A) Quantitative LDA analysis of the Pi and PPi concentrations (0-130 μM) shows smooth saturation behavior. (Panel B) Quantitative linear regression analysis of Pi concentrations using the support vector machine (SVM) algorithm. An unknown Pi concentration shown as a red dot (●) was predicted with 2.3% error. (Panel C) Quantitative linear regression analysis of PPi concentrations using support vector machine (SVM) shows the correct prediction of an unknown concentration (●) with a prediction of 4.8%.

A

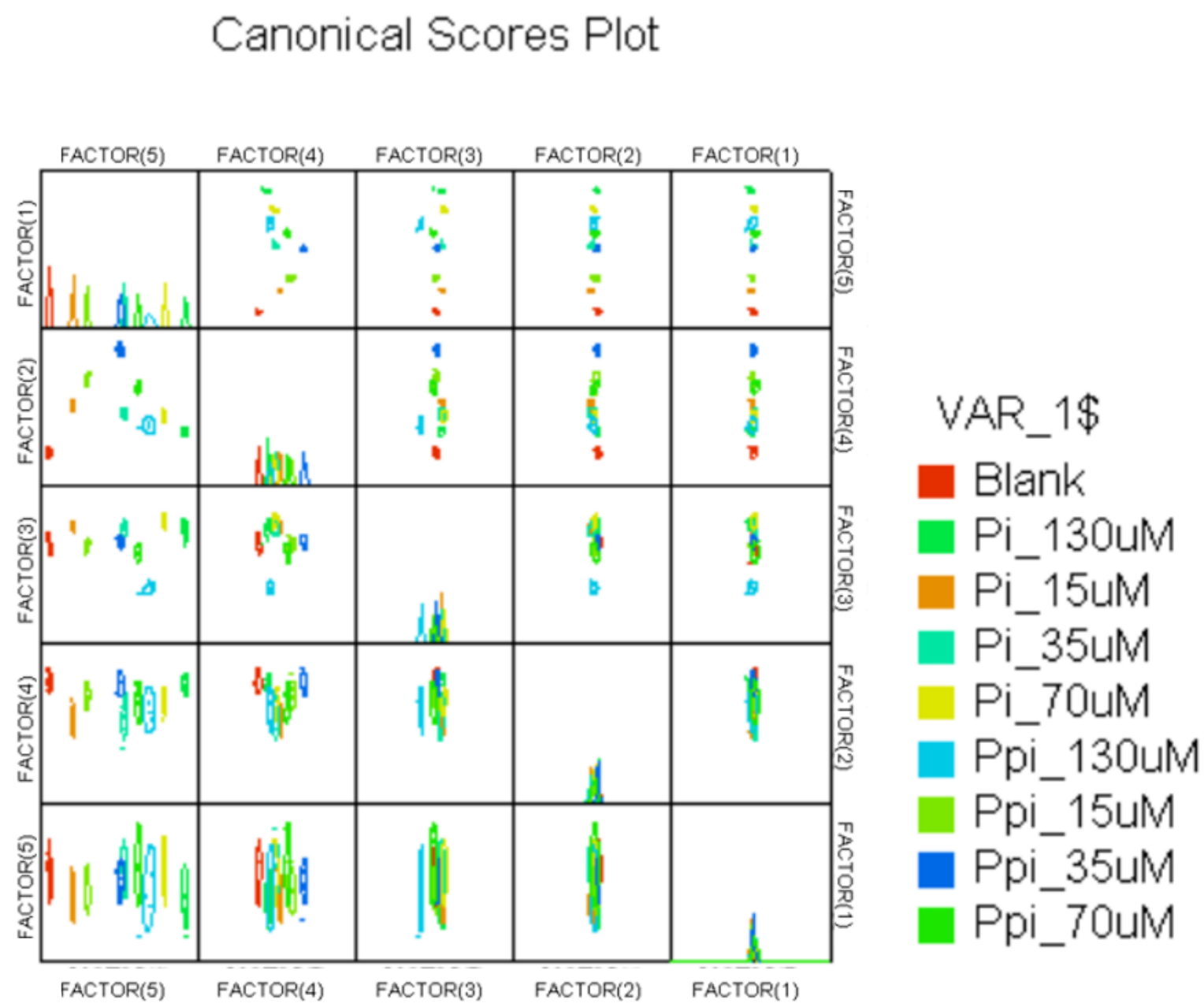


B

**Jackknifed Classification Matrix**

| | Blank | Pi_130uM | Pi_15uM | Pi_35uM | Pi_70uM | Ppi_130uM | Ppi_15uM | Ppi_35uM | Ppi_70uM | %correct |
|---|---|---|---|---|---|---|---|---|---|---|
| Blank | 20 | 0 | 0 | 0 | 0 | 0 | 0 | 0 | 0 | 100 |
| Pi_130uM | 0 | 20 | 0 | 0 | 0 | 0 | 0 | 0 | 0 | 100 |
| Pi_15uM | 0 | 0 | 20 | 0 | 0 | 0 | 0 | 0 | 0 | 100 |
| Pi_35uM | 0 | 0 | 0 | 20 | 0 | 0 | 0 | 0 | 0 | 100 |
| Pi_70uM | 0 | 0 | 0 | 0 | 20 | 0 | 0 | 0 | 0 | 100 |
| Ppi_130uM | 0 | 0 | 0 | 0 | 0 | 18 | 0 | 0 | 0 | 100 |
| Ppi_15uM | 0 | 0 | 0 | 0 | 0 | 0 | 20 | 0 | 0 | 100 |
| Ppi_35uM | 0 | 0 | 0 | 0 | 0 | 0 | 0 | 20 | 0 | 100 |
| Ppi_70uM | 0 | 0 | 0 | 0 | 0 | 0 | 0 | 0 | 19 | 100 |
| Total | 20 | 20 | 20 | 20 | 20 | 18 | 20 | 20 | 19 | 100 |

**Figure S28**: LDA canonical scores plot (A) and Jackknifed Classification matrix (B) for array of sensors 1 and 6, and its complexes with different concentrations of Pi and PPi.

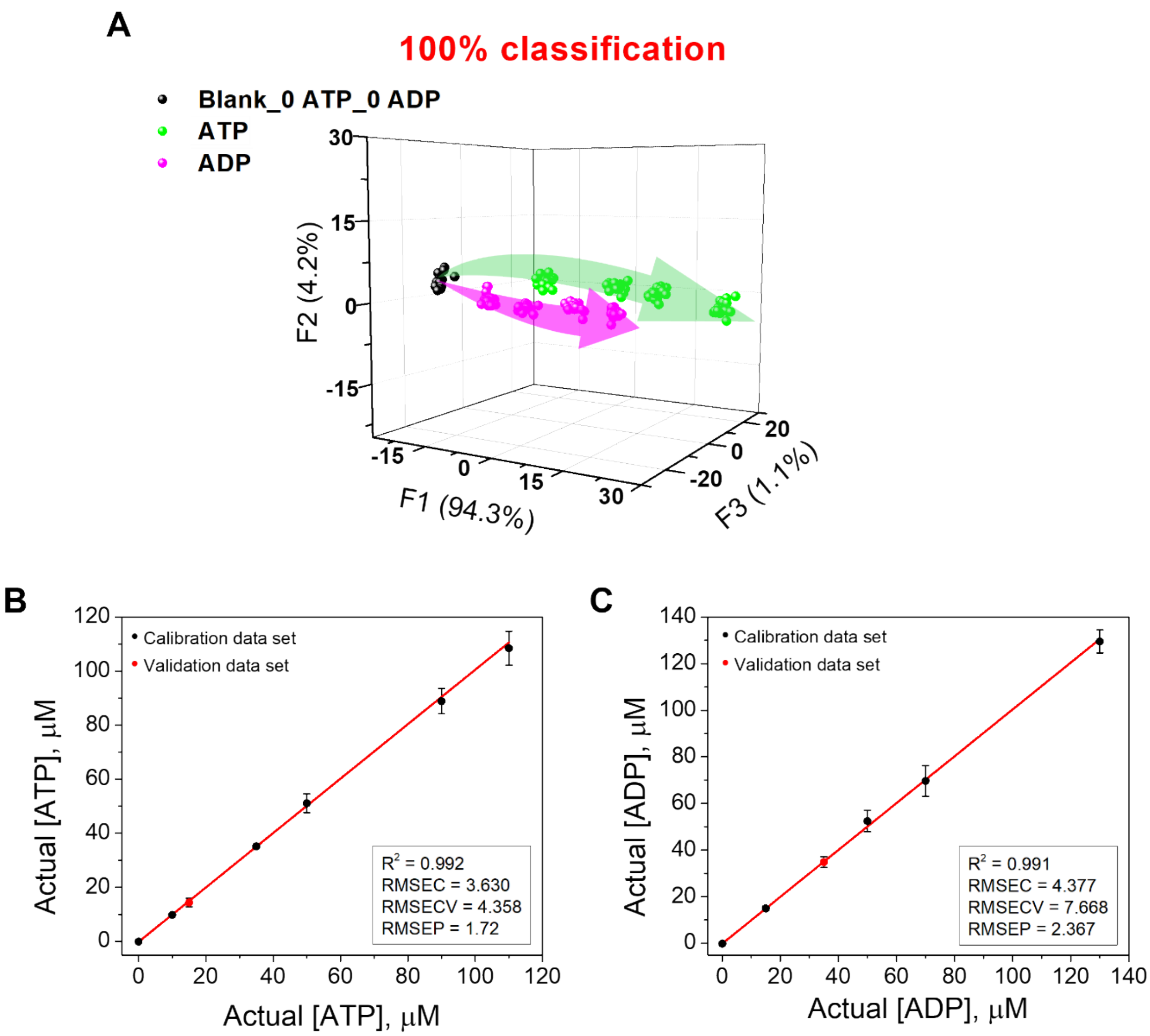


**Figure S29**: Quantitative analysis of ATP and ADP from array of sensors **1** and **6**. (Panel A) Quantitative LDA analysis of the Pi and PPi concentrations (0-130 μM) shows smooth saturation behavior. (Panel B) Quantitative linear regression analysis of ATP concentrations using the support vector machine (SVM) algorithm. An unknown ATP concentration shown as a red dot (●) was predicted with 1.7% error. (Panel C) Quantitative linear regression analysis of ADP concentrations using support vector machine (SVM) shows the correct prediction of an unknown concentration (●) with a prediction of 2.3%.

**A**

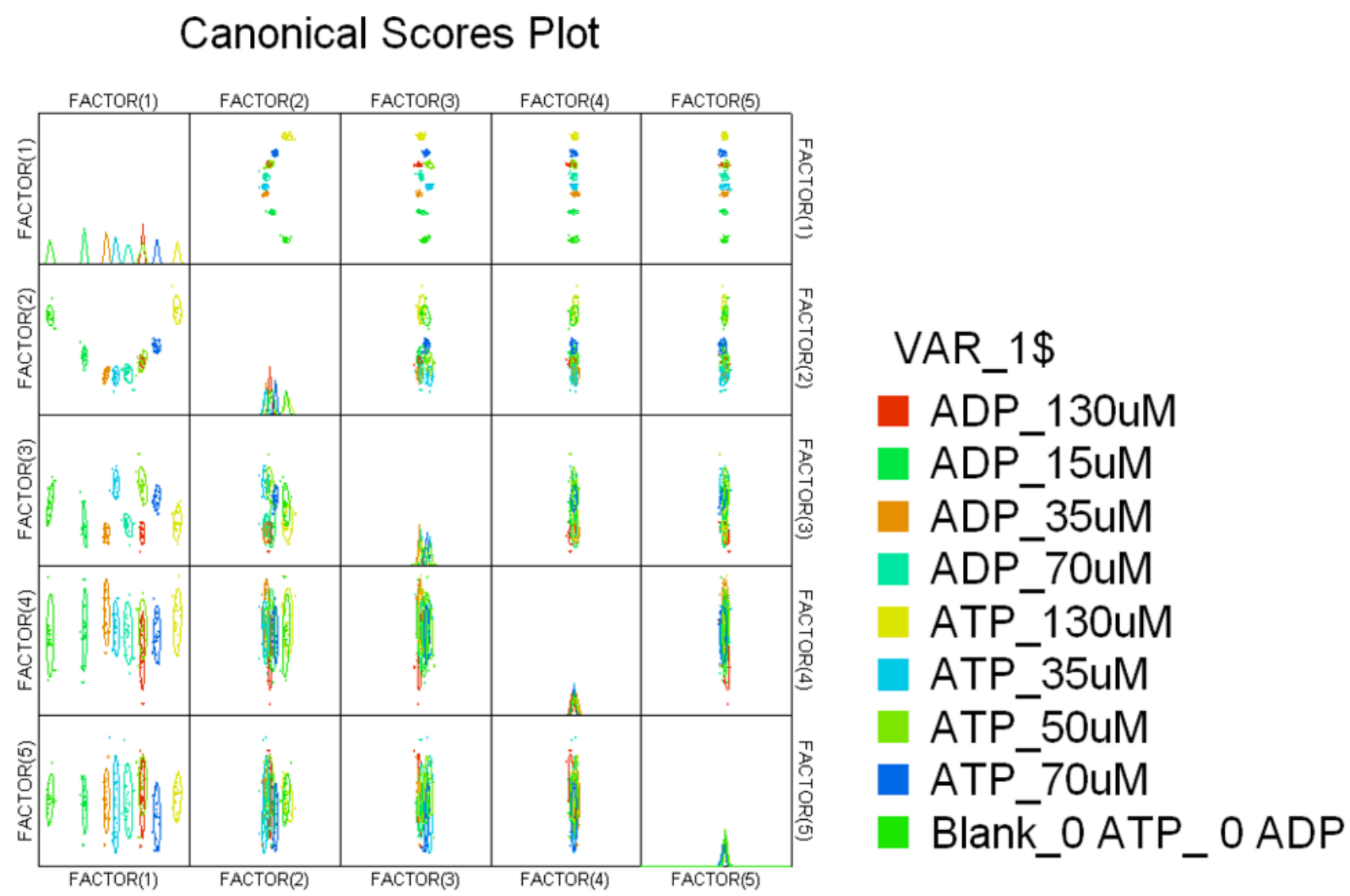


**B**

**Jackknifed Classification Matrix**

| | ADP_130uM | ADP_15uM | ADP_35uM | ADP_70uM | ATP_130uM | ATP_35uM | ATP_50uM | ATP_70uM | Blank_0 ATP_ 0 ADP | %correct |
|---|---|---|---|---|---|---|---|---|---|---|
| ADP_130uM | 20 | 0 | 0 | 0 | 0 | 0 | 0 | 0 | 0 | 100 |
| ADP_15uM | 0 | 20 | 0 | 0 | 0 | 0 | 0 | 0 | 0 | 100 |
| ADP_35uM | 0 | 0 | 20 | 0 | 0 | 0 | 0 | 0 | 0 | 100 |
| ADP_70uM | 0 | 0 | 0 | 20 | 0 | 0 | 0 | 0 | 0 | 100 |
| ATP_130uM | 0 | 0 | 0 | 0 | 20 | 0 | 0 | 0 | 0 | 100 |
| ATP_35uM | 0 | 0 | 0 | 0 | 0 | 20 | 0 | 0 | 0 | 100 |
| ATP_50uM | 0 | 0 | 0 | 0 | 0 | 0 | 20 | 0 | 0 | 100 |
| ATP_70uM | 0 | 0 | 0 | 0 | 0 | 0 | 0 | 20 | 0 | 100 |
| Blank_0 ATP_ 0 ADP | 0 | 0 | 0 | 0 | 0 | 0 | 0 | 0 | 20 | 100 |
| Total | 20 | 20 | 20 | 20 | 20 | 20 | 20 | 20 | 20 | 100 |

**Figure S30**: LDA canonical scores plot (A) and Jackknifed Classification matrix (B) for array of sensors **1** and **6**, and its complexes with different concentrations of ATP and ADP.

## Limit of Detection and Limit of Quantitation Determination

The limit of detection (LOD) and Limit of Quantification were determined from a linear regression analysis of the quantitative HT data obtained from the BMG CLARIOstar microplate reader.

The following equations were used for calculations, where σ represents the standard difference and m refers to the slope of the line:

LOD = |(3.3×σ)/m|

LOQ = |10×σ)/m|

Sensor **1** with Pi: The data showed good linearity ($R^2$ ~0.994) from 0 to 40.0 $\mu$M of Pi, where the average of 20 replicas of the control resulted in σ = 196.44 and the average slope of 20 replicas for all data points from 0 - 40.0 $\mu$M of Pi was m = $6.90\times10^{8}$. Therefore, the LOD was calculated to be 0.94 $\mu$M and the LOQ was calculated to be 2.84 $\mu$M.

Sensor **1** with PPi: The data showed good linearity ($R^2$ ~0.989) from 0 to 35.0 $\mu$M of PPi, where the average of 20 replicas of the control resulted in σ = 246.42 and the average slope of 20 replicas for all data points from 0 - 35.0 $\mu$M of PPi was m = $7.86\times10^{8}$. Therefore, the LOD was calculated to be 1.03 $\mu$M and the LOQ was calculated to be 3.13 $\mu$M.

Sensor **1** with ATP: The data showed good linearity ($R^2$ ~0.991) from 0 to 45.0 $\mu$M of ATP, where the average of 20 replicas of the control resulted in σ = 595.59 and the average slope of 20 replicas for all data points from 0 - 45.0 $\mu$M of ATP was m = $6.47\times10^{8}$. Therefore, the LOD was calculated to be 3.03 $\mu$M and the LOQ was calculated to be 9.19 $\mu$M.

Sensor **1** with ADP: The data showed good linearity ($R^2$ ~0.995) from 0 to 50.0 $\mu$M of ADP, where the average of 20 replicas of the control resulted in σ = 597.70 and the average slope of 20 replicas for all data points from 0 - 50.0 $\mu$M of ADP was m = $4.41\times10^{8}$. Therefore, the LOD was calculated to be 4.47 $\mu$M and the LOQ was calculated to be 13.53 $\mu$M.

Sensor **6** with Pi: The data showed good linearity ($R^2$ ~0.992) from 0 to 45.0 $\mu$M of Pi, where the average of 20 replicas of the control resulted in σ = 161.15 and the average slope of 20 replicas for all data points from 0 - 45.0 $\mu$M of Pi was m = $5.34\times10^8$. Therefore, the LOD was calculated to be 0.99 $\mu$M and the LOQ was calculated to be 3.01 $\mu$M.

Sensor **6** with PPi: The data showed good linearity ($R^2$ ~0.990) from 0 to 70.0 $\mu$M of PPi, where the average of 20 replicas of the control resulted in σ = 78.89 and the average slope of 20 replicas for all data points from 0 - 70.0 $\mu$M of PPi was m = $3.79\times10^8$. Therefore, the LOD was calculated to be 0.68 $\mu$M and the LOQ was calculated to be 2.08 $\mu$M.

Sensor **6** with ATP: The data showed good linearity ($R^2$ ~0.991) from 0 to 70.0 $\mu$M of ATP, where the average of 20 replicas of the control resulted in σ = 374.61 and the average slope of 20 replicas for all data points from 0 - 70.0 $\mu$M of ATP was m = $3.46\times10^8$. Therefore, the LOD was calculated to be 3.57 $\mu$M and the LOQ was calculated to be 10.82 $\mu$M.

Sensor **6** with ADP: The data showed good linearity ($R^2$ ~0.987) from 0 to 70.0 $\mu$M of ADP, where the average of 20 replicas of the control resulted in σ = 122.20 and the average slope of 20 replicas for all data points from 0 - 70.0 $\mu$M of ADP was m = $2.51\times10^8$. Therefore, the LOD was calculated to be 1.60 $\mu$M and the LOQ was calculated to be 4.86 $\mu$M.

## Complex formation: Evidence from ESI-MS

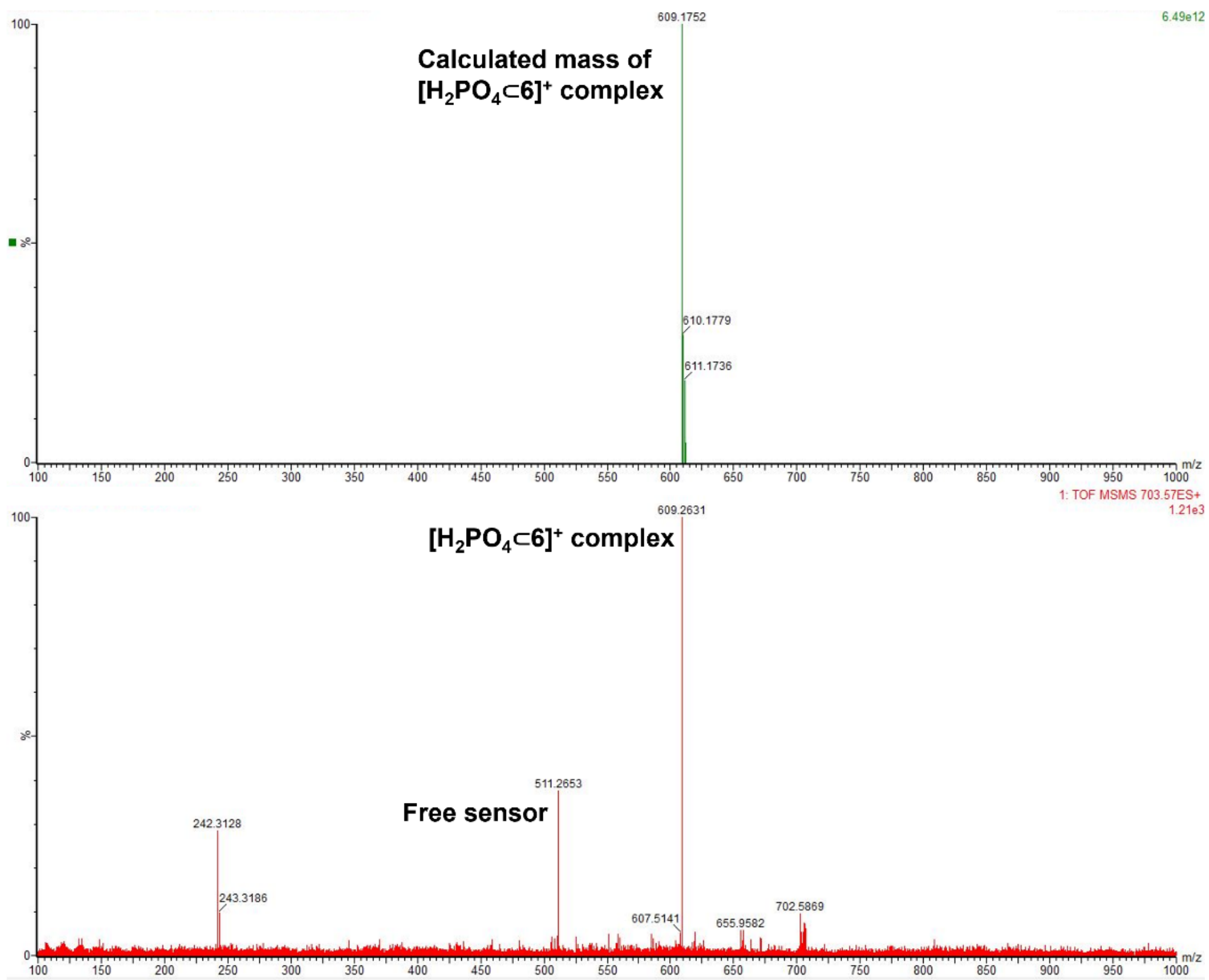


**Figure S31**: ESI-MS spectrum of $[H_2PO_4$⊂$6]^+$ obtained using isothiouronium **6** and an excess of $TBAH_2PO_4$ (circa 15 eq) in $CHCl_3$/MeOH (8/2:v/v).

## Macrocyclic vs Linear sensors comparison

**Table S1. Binding constants ($K_{assoc}$, $M^{-1}$)[a] calculated for sensors 3[5], 1, 6, and 7 and the sodium salts of anionic analytes.**

| Anion/Sensor | **3** Macrocycle [b] | **1** Ammonium | **6** Isothiouronium | **7** Guanidinium |
|---|---|---|---|---|
| $H_2PO_4^-$ | $1.65\times10^6$ | $3.87\times10^4$ | $1.44\times10^5$<br>$8.82\times10^4$ | $8.00\times10^3$ |
| $H_2P_2O_7^{2-}$ | $5.82\times10^5$ | $5.43\times10^4$ | $5.35\times10^4$ [b] | $2.81\times10^4$ |
| $HAsO_4^{2-}$ | $3.55\times10^6$ | $2.23\times10^4$ | $2.21\times10^3$<br>$3.14\times10^4$ | $1.14\times10^4$ |
| $AMP^-$ | $1.61 \times 10^5$ | <100 | <100 | <100 |
| $ADP^{2-}$ | <100[c] | $1.50\times10^4$ | $1.37\times10^4$ | $2.83\times10^3$ |
| $ATP^{3-}$ | <100[c] | $2.57\times10^4$ | $1.41\times10^4$ | <100 |
| $GlyP^{2-}$ | $2.70 \times 10^5$ | $1.54\times10^3$ [b] | $2.81\times10^3$ [b] | <100 |
| $NO_3^-$ | NR | NR | NR | NR |
| $F^-$ | ND | <50 | <50 | <50 |
| $Cl^-$ | <10 | <50 | <50 | NR |

[a] The $K_{assoc}$ values were calculated from the change in fluorescence intensity upon incremental addition of each anion (sodium salts in water) to a 15 μM solution of the sensors in water-DMSO (1:9 v/v, pH 5.5 for **1**, **6**, and **7**) and 5 μM solution of the sensor **3** in water-DMSO (1.5:8.5 v/v, pH 6.8 for **3**). For sensors **1** and **6**, the excitation/emission wavelengths were 350/548 nm; for **7**, 400/548 nm and for **3**, 370/548 nm. [b] The fluorescence spectrometer was used to acquire data. [c] $K_{assoc}$ was not determined due to the biphasic nature of the isotherm. ND - not determined due to what appears to be a partial deprotonation of the sensor and the ensuing complex equilibria. NR - no appreciable response was observed.

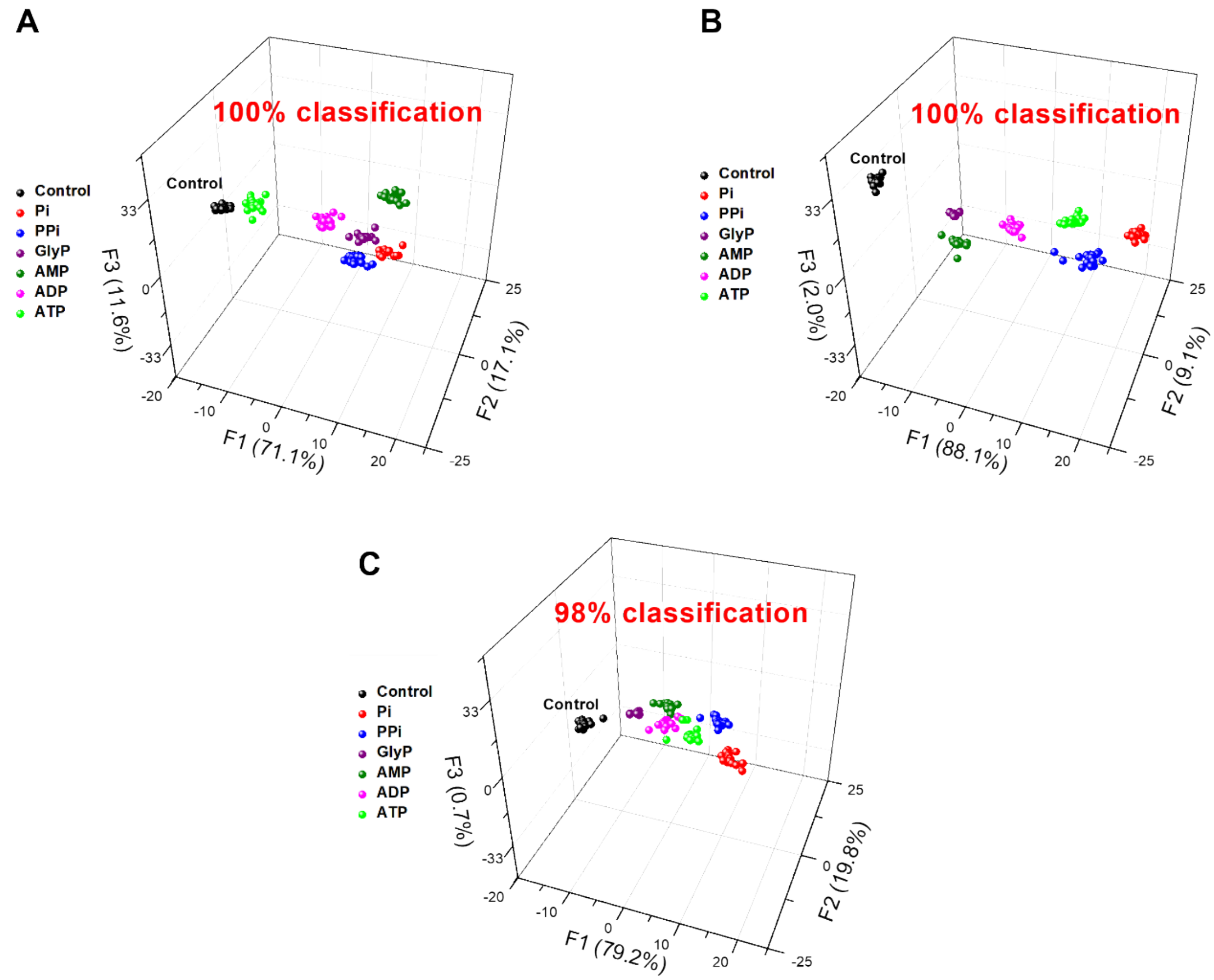


**Figure S32**: 3D graphical output of qualitative LDA displaying clusters of 6 analytes and 1 control from (**A**) macrocycle **3** and (**B**) an array of sensors **1** and **6** (**C**) isothiouronium **6** (1:3 Host:Guest).

## X-ray Crystallography

Diffraction data were collected at 120 K with a Bruker Duo diffractometer equipped with an APEX II CCD detector using Cu Kα radiation. The cell constants were determined from the full data set, integrated with SAINT V8.40A (Bruker (2012). *SAINT V8.40A*. Bruker AXS Inc., Madison, Wisconsin, USA.) and an absorption and scaling were performed by SADABS-2016/2 (Bruker (2001). *SADABS 2016/2.* Bruker AXS Inc., Madison, Wisconsin, USA.). The structure was solved by the dual-space method with SHELXT 2014/5 (Sheldrick, G. M. (2015). *Acta Cryst.* A**71**, 3-8.) and refined against $F^2$ by the least squares method with SHELXL 2018/3 (Sheldrick, G. M. (2015). *Acta Cryst.* C**71**, 3-8.). All non-H atoms were refined with anisotropic displacement parameters, with the exception of the disordered lattice acetonitrile, which was treated as a two component disorder and modeled with isotropic displacement parameters. Hydrogen atoms involved in hydrogen-bonding ($RNH_3^+$, $HPO_4^{2-}$ and $H_2O$) were located from the Fourier difference map and refined isotropic displacement parameters. Other hydrogen atoms were calculated on idealized positions and refined with a riding model.

Table S1. Crystal data, data collection and refinement details of sensor **1**.

| | |
|---|---|
| $C_{18}H_{32}N_5O_7PS$ | Volume, V = 4591.1 (3) $Å^3$ |
| $M_r$ = 493.51 | Z = 8 |
| Cell parameters: | Radiation, Cu-Kα radiation |
| a = 9.0693 (3) Å | μ = 2.349 $mm^{-1}$ |
| b = 11.4759 (4) Å | Temperature, T = 120 (1) K |
| c = 44.1121 (16) Å | Density, □ = 1.428 g $cm^{-3}$ |
| α = 90° | |
| □ = 90° | |
| γ = 90° | |
| Diffractometer Bruker Apex Duo | $θ_{max}$ = 70.0° |
| ω, φ scan | h = -10 → 10 |
| 24513 measured reflections | k = -13 → 13 |
| 4329 independent reflections | l = -52 → 53 |
| 3787 reflections with I > 2σ(I ) | |
| $R_{int}$ = 0.087 | |
| Refinement on $F^2$ | Mixture of independent and constrained H-atom refinement |
| $R_1$[a] (F, I > 2σ(I )) = 0.0552 | Calculated weights w = $1/[σ^2(Fo^2) + (0.0237P)^2 + 11.7421P]$ where $P = (Fo^2 + 2Fc^2)/3$ |
| $wR_2$[b] ($F^2$, all data) = 0.1336 | (Δ/σ)max <0.0001 |
| Goodness of fit, S = 1.26 | $□_{rmax}$ = 0.491 $eÅ^{-3}$ |
| 4329 reflections | $□_{rmin}$ = -0.379 $eÅ^{-3}$ |
| 326 parameters | |

[a] $R_1 = Σ||F_o| - |F_c||/Σ|F_o|$. [b] $wR_2 = [Σ[w(F_o^2 - F_c^2)^2]/Σ[w(F_o^2)^2]]^{1/2}$.

## References

1. A. Radujević, A. Penavić, R. Z. Pavlović, J. D. Badjić and P. Anzenbacher, Jr., *Chem*, **2022**, *8*, 2228-2244.

2. R. A. Batey and D. A. Powell, *Org. Lett.*, **2000**, *2*, 3237-3240.

3. S. Kang and T. H. Kim, *Tetrahedron Lett.*, **2021**, *84*, 153450.

4. M. C. Nielsen, J. Borch and T. Ulven, *Bioorg. Med. Chem.*, **2009**, *17*, 8241-8246.

5. A. R. Sartori, A. Radujević, S. M. George and P. Anzenbacher, Jr., *Chem. Sci.*, **2023**, *14*, 7545-7552.
